# Cost-effectiveness of posterior versus anterior surgery for cervical radiculopathy: results from a multicentre randomised non-inferiority trial (FACET)

**DOI:** 10.1007/s00586-024-08340-4

**Published:** 2024-06-07

**Authors:** A. E. H. Broekema, N. F. Simões de Souza, R. J. M. Groen, R. Soer, M. F. Reneman, J. M. A. Kuijlen, A. D. I. van Asselt

**Affiliations:** 1 Department of Neurosurgery, University Medical Centre Groningen, University of Groningen, Postal Box 30.001, 9700 RB Groningen, The Netherlands; 2 Department of Anaesthesiology, Groningen Pain Centre, University Medical Centre Groningen, University of Groningen, Groningen, The Netherlands; 3 Research Group Smart Health, Saxion University of Applied Sciences, Enschede, The Netherlands; 4 Department of Rehabilitation, University Medical Centre Groningen, University of Groningen, Groningen, The Netherlands; 5 Department of Epidemiology, University Medical Centre Groningen, University of Groningen, Groningen, The Netherlands; 6 Department of Health Sciences, University Medical Centre Groningen, University of Groningen, Groningen, The Netherlands

**Keywords:** Anterior cervical discectomy and fusion, Cervical radiculopathy, Cost-effectiveness, Health economics, Posterior cervical foraminotomy, Randomised controlled trial

## Abstract

**Purpose:**

For cervical nerve root compression, anterior cervical discectomy with fusion (anterior surgery) or posterior foraminotomy (posterior surgery) are safe and effective options. Posterior surgery might have a more beneficial economic profile compared to anterior surgery. The purpose of this study was to analyse if posterior surgery is cost-effective compared to anterior surgery.

**Methods:**

An economic evaluation was performed as part of a multicentre, noninferiority randomised clinical trial (Foraminotomy ACDF Cost-effectiveness Trial) with a follow-up of 2 years. Primary outcomes were cost-effectiveness based on arm pain (Visual Analogue Scale (VAS; 0–100)) and cost-utility (quality adjusted life years (QALYs)). Missing values were estimated with multiple imputations and bootstrap simulations were used to obtain confidence intervals (CIs).

**Results:**

In total, 265 patients were randomised and 243 included in the analyses. The pooled mean decrease in VAS arm at 2-year follow-up was 44.2 in the posterior and 40.0 in the anterior group (mean difference, 4.2; 95% CI, − 4.7 to 12.9). Pooled mean QALYs were 1.58 (posterior) and 1.56 (anterior) (mean difference, 0.02; 95% CI, − 0.05 to 0.08). Societal costs were €28,046 for posterior and €30,086 for the anterior group, with lower health care costs for posterior (€12,248) versus anterior (€16,055). Bootstrapped results demonstrated similar effectiveness between groups with in general lower costs associated with posterior surgery.

**Conclusion:**

In patients with cervical radiculopathy, arm pain and QALYs were similar between posterior and anterior surgery. Posterior surgery was associated with lower costs and is therefore likely to be cost-effective compared with anterior surgery.

**Supplementary Information:**

The online version contains supplementary material available at 10.1007/s00586-024-08340-4.

## Introduction

With an ever growing aging population, the prevalence of cervical spinal degeneration leading to symptomatic nerve root compression is expected to increase. Patients with cervical nerve root compression can present with disabling arm pain, with or without neurological deficits [1, 2]. They are first treated conservatively, but if this fails surgical treatment can be considered. For patients with a one-sided, one-level nerve root compression, without spinal cord compression, anterior cervical discectomy with fusion (anterior surgery) and posterior cervical foraminotomy (posterior surgery) are both viable options with similar clinical outcomes [3–5].

Cost and value of health care are of global importance as health care expenditure is continuously rising [6]. Anterior surgery is often accompanied by costly intervertebral implants, and is associated with a longer procedural length [3]. Therefore, posterior surgery might have a more beneficial economic profile. This is also supported by the currently available evidence, although based on retrospective studies or small not-generalisable cohorts [7–10]. Therefore, an evaluation of the cost-effectiveness of posterior versus anterior surgery in a prospective, randomised controlled trial was needed.

The Foraminotomy ACDF Cost-Effectiveness Trial is a multicentre randomised controlled trial demonstrating noninferior clinical outcome of posterior surgery compared to anterior surgery [3, 5, 11]. Posterior surgery was hypothesised to have lower costs compared to anterior surgery. In the present study, cost-effectiveness and cost-utility were analysed after 2 years of follow-up.

## Methods

### Trial design

The FACET is a randomised, multicentre, noninferiority trial including participants treated with posterior or anterior surgery for cervical radiculopathy due to single-level one-sided foraminal nerve root compression. Full details on the inclusion, randomisation, surgical techniques, health economic analysis plan as well as clinical outcome have been published previously [3, 5, 11]. FACET hypothesised that posterior surgery would be cost-effective, and would have lower direct and indirect costs in comparison with anterior surgery. In the reporting of this study, the Consolidated Health Economic Evaluation Reporting Standards (CHEERS) were used [12]. The study was approved by the research ethical board of the University Medical Center Groningen, The Netherlands. All patients provided written informed consent before randomisation. A Dutch patient group (ZorgBelang Groningen) was actively involved in the design of the study.

### Study population

**Fig. 1:**
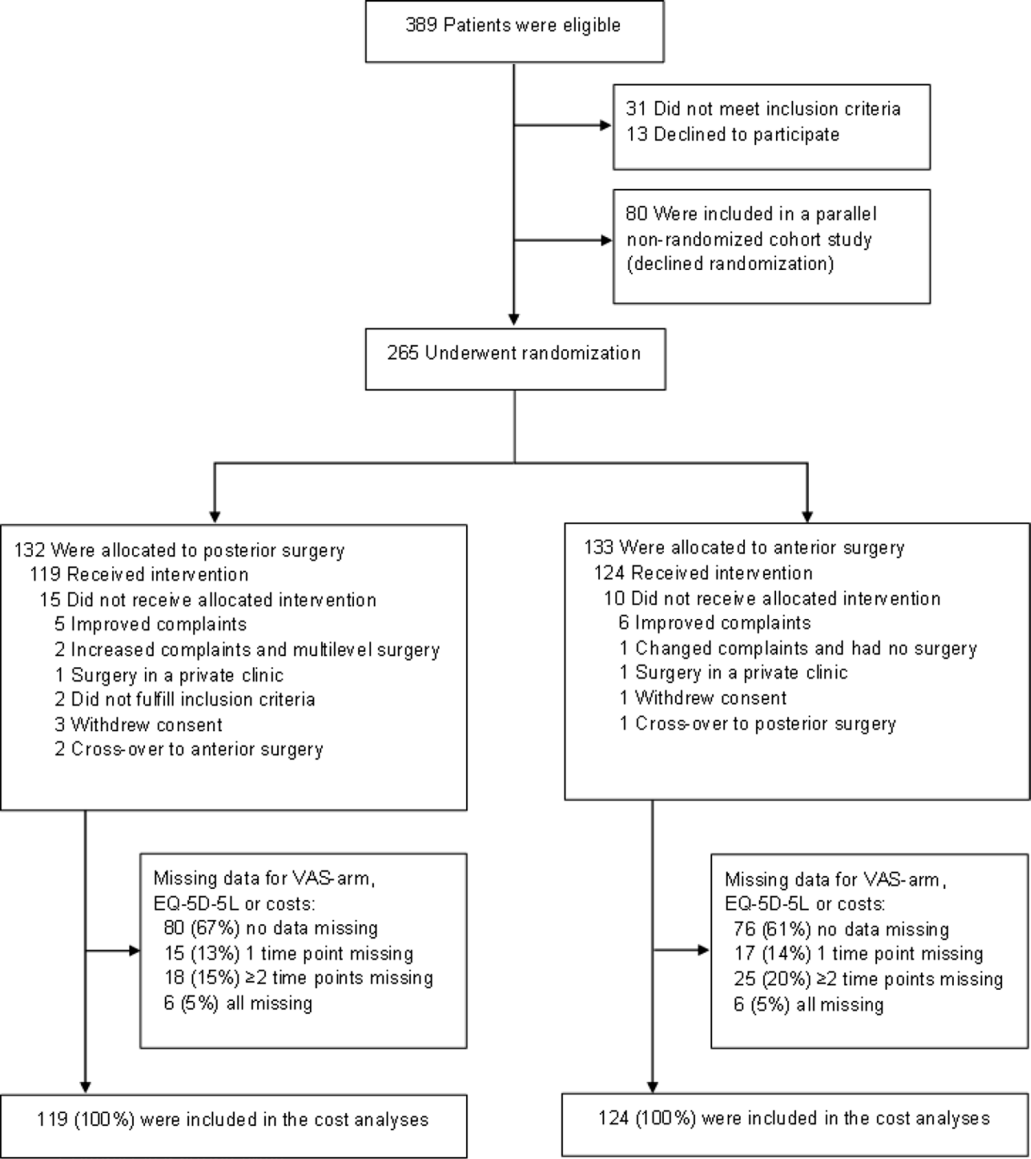
Flow-chart of randomised patients, their primary treatment and follow-up status. Abbreviations: Visual Analogue Scale (VAS) for arm pain, EuroQol 5-Dimensions 5-Level questionnaire (EQ-5D-5L), Participants received web-based questionnaires (assessing patient reported outcome measures, medical consumption and productivity loss) 6 weeks after surgery and every six months until 2 years after surgery

In the FACET, 265 participants were randomised, of whom 243 received the allocated treatment (see Fig. 1). The most common reason to not receive the allocated treatment was spontaneous improvement of symptoms (n = 11). Participants were included from nine hospitals in the Netherlands, of which two were academic teaching hospitals and seven large regional hospitals. At six weeks and thereafter every six months up until two years after surgery, patients received a web-based questionnaire with multiple patient reported outcome measures (PROMs) including questionnaires about medical consumption and productivity losses [11].

### Primary outcomes

Cost-effectiveness was analysed alongside the trial, taking a societal perspective with a time horizon of two years, equivalent to the duration of the FACET. The societal perspective includes all costs related to health care resource use and productivity losses. According to Dutch pharmaco-economic guidelines [13], discounting was applied for costs (4%) and effects (1.5%) in the second year to unify costs and effects occurring in different years.

Two primary outcome measures were defined, resulting in two incremental cost-effectiveness ratios (ICERs) for posterior compared to anterior surgery [11]. An ICER is the difference in costs between two interventions, divided by the difference in their (clinical) effect. First, incremental costs for relief in arm pain were calculated, based on the area under the curve for arm pain (Visual Analogue Scale (VAS; 0–100 mm)).

Secondly, incremental costs per Quality Adjusted Life Year (QALY) gained were calculated using the EuroQol 5-Dimensions 5-Level questionnaire (EQ-5D-5L) [14]. The EQ-5D-5L scores were converted into utility scores (− 0.446—1) based on the Dutch tariffs [15]. QALYs were calculated by multiplying utility scores by time spent in each health state.

### Secondary outcomes

Cost-effectiveness (based on VAS-arm) and cost-utility (based on QALYs) from a health care perspective, meaning all costs related to health care resource use without costs related to productivity losses, were considered secondary outcomes.

### Measurement and valuation of healthcare resource use and lost productivity

The costs of healthcare resource use were derived from the Institute for Medical Technology Assessment Medical Consumption Questionnaire [16] (iMCQ) by multiplying visits to caregivers, procedures performed, clinical admissions, medication use, and other healthcare consumption by standard unit prices as recommended in the Dutch Costing Manual [17]. If necessary, prices were converted to the 2020 price level using the price index provided by the Dutch Central Bureau of Statistics [18]. Pain medication costs were based on generic prices from the Dutch “Pharmacotherapeutic Compass” [19]. Centre-specific surgical costs were drawn from the Dutch ‘diagnosis treatment combination’ (DBC) system, which is similar to the ‘diagnosis related groups’ (DRG) billing system used in many European countries, and included prices for implants, microscopes and haemostatics [20]. Admission days and visits to the outpatient clinic, related to the primary intervention, were separately calculated (in contrast to using the cost price for the DBC, which already includes the average costs for hospital admission and outpatient clinic visits).

Productivity losses were assessed with the validated Institute for Medical Technology Assessment Productivity Cost Questionnaire (iPCQ) [21, 22]. For productivity losses in paid work, absenteeism (complete absence from work) as well as presenteeism (productivity loss during work) were distinguished. Lost productivity was valued using an average hourly wage taken from the Dutch guidelines. From this guideline, also a shadow price for unpaid productivity was used [13].

The ‘friction cost method’ was used, assuming that after a period of 85 days of complete absence, a worker got replaced by another worker, and productivity would be restored [23].

The recall period, meaning the time period that is questioned, was three months for the iMCQ and one month for the iPCQ. Therefore, values of measurements were multiplied according to the questioned time period (see Supplementary Information, Table 1). All costs were expressed in Euros.

### Statistical analysis

Baseline characteristics, including education level and work status, were described as means with standard deviation (SD), or median and interquartile range, depending on the distribution of the variable. Between-group differences were tested with an independent t test, Mann–Whitney U, or Pearson χ2 depending on level of measurement and distribution of data.

Regarding patient reported outcomes, the area under the curve was calculated using all time points.

Missing data in costs and patient reported outcomes were handled by performing multiple imputation with five imputation sets [25, 26]. Five imputation sets are generally considered sufficient, unless rates of missing data are very high [27]. Data were assumed to be missing at random. Variables included in the multiple imputation procedure were baseline and follow-up measurements of VAS-arm, EQ-5D-5L utility score, NDI, and variables on annual healthcare resource use per category (e.g. general physician visits, inpatient days, outpatient visits) and productivity loss. In addition, age and gender were included as covariates. For each imputation set, cost-effectiveness was bootstrapped separately with 5,000 resamples and data were reported as pooled means with bootstrapped 95% confidence intervals (CI) [28]. Results of all analyses were presented in incremental cost-effectiveness planes (ICEPs) and cost-effectiveness acceptability curves (CEACs). CEACs visualise the probability that an intervention is cost-effective, given a certain societal willingness-to-pay for a millimetre decrease in arm pain or for one QALY gained. For the Netherlands, this willingness-to-pay threshold is 20.000–80.000 Euros depending on burden of disease [29].

Sensitivity analyses were performed by comparing the results of the primary analyses with similar analyses in complete cases only. Furthermore, cost-effectiveness analysis with the VAS arm pain as effect was compared to analyses using proportion of success based on the Odom criteria (4-point rating scale, ‘excellent’ and ‘good’ considered as successful) [30, 31] as well as the Neck Disability Index (NDI) [32, 33] as treatment effect. For the dichotomised Odom criteria, available data at 2 year follow-up was used, as it was not possible to perform reliable multiple imputations. The Odom criteria is, together with the VAS arm score, the primary outcome of the clinical trial. SPSS software (version 28.0, IBM Corp., Armonk, NY) was used for data analysis and Microsoft Excel 365 for bootstrapping simulation. The detailed statistical analysis plan is presented in Supplementary File 1.

## Results

### Participants

Characteristics of the participants, including education level and work status are presented in Table 1. Detailed characteristics of the participants have been published previously [3, 5]. Information regarding health care resource use and productivity losses at baseline are presented in the Supplementary Information, Table 2.

**Table 1:** Baseline characteristics of included participants.

	No. (%)	
	Posterior surgery n = 119	Anterior surgery n = 124*
Age, mean (SD), y	51.6 (8.5)	51.0 (8.3)
*Sex*		
Female	66 (55)	58 (47)
Male	53 (45)	66 (53)
Body Mass Index, median (IQR)^a^	27 (24–30)	27 (24–30)
Symptom duration, median (IQR), wk	34 (26–52)	32 (20–52)
*ASA classification*^*b*^		
ASA I	55 (46)	66 (53)
ASA II	59 (50)	53 (43)
ASA III	5 (4)	5 (4)
Current smoker	53 (46)	47 (39)
Use of NSAIDS	39 (33)	35 (29)
*Highest education*^*c*^		
None or primary school	7 (6)	7 (6)
High school or equivalent	38 (34)	49 (42)
Vocational education	39 (35)	37 (32)
Bachelor degree/Pre-university	22 (20)	20 (17)
University or higher	6 (5)	3 (3)
*Daily occupation*		
Paid job	81 (72)	90 (78)
Unpaid job	6 (5)	8 (7)
Job seeking	3 (3)	4 (3)
Unemployed due to health issues	15 (13)	5 (4)
Retired	7 (6)	9 (8)
Sick leave before surgery	45 (56)	55 (61)
Duration of sick leave, day, median (IQR)	85 (13–156)	66 (16–134)

^a^Body Mass Index is the weight in kilograms divided by the square of the height in meters

^b^The classification system of ASA ranges from I to VI, where higher classes indicate a greater risk for perioperative complications. No patients had an ASA IV,V or VI classification

^c^The Dutch educational system has 8 years of primary education, starting from the age of 4, followed by 4–6 years of secondary education. After 4 years of secondary education, vocational education is possible, with a focus on practical skills. After 5 years of secondary education, a pre-university trajectory is possible leading to a bachelor’s degree (4 years). After 6 years of secondary education one can enter directly into university leading to a bachelor’s degree (3 years) or master’s degree (1–2 additional years)

ASA: American Society of Anaesthesiologists classification, SD: Standard deviation, IQR: Inter Quartile Range, No. (number of patients), NSAID: Non-Steroidal Anti-Inflammatory Drug

*Percentages may not total 100, because of rounding. Data was missing for educational level and work status in 7 participants in the posterior surgery group and 7 in the anterior group

### Clinical outcomes and costs

Clinical outcome at two years follow-up are presented in Table 2. Total costs were €28,046 for posterior surgery versus €30,086 for anterior surgery, with a pooled mean difference of €− 2674 (95% bootstrapped CI, −14,953 to 4231). Cost differentiation is presented in Table 3, detailed information on number of patients reporting for each subcategory are presented in the Supplementary Information, Table 3.

**Table 2:** Clinical outcome at two year follow-up

	Posterior surgery	Anterior surgery	Difference (95% CI)*
	n = 119	n = 124	
VAS arm pain, 0 to 100	44.2	40.0	4.2 (− 4.65 to 12.9)
QALYs, − 0.892 to 2	1.58	1.56	0.02 (− 0.05 to 0.08)
NDI, 0 to 100	42.6	43.2	− 0.6 (− 7.3 to 5.9)
Successful Odom^a^, 0 to 1	0.78	0.81	− 0.03 (− 0.16 to 0.09)

^a^‘Successful Odom’ is the proportion of patients graded ‘excellent’ or ‘good’ on the modified Odom criteria, based on patients with complete cost data for all time points and an available Odom score (n = 80 in the posterior group and n = 73 in the anterior group)

*All values are presented as pooled means with bootstrapped confidence intervals, with exception of the Successful Odom score. The Odom score is expressed as proportions per group and difference in proportions between groups

CI: Confidence Interval, VAS: Visual Analogue Scale, QALYs: Quality Adjusted Life Years, NDI: Neck Disability Index

**Table 3:** Cost differentiation

	Posterior surgery n = 119	Anterior surgery n = 124	Difference (95% CI)*
Surgery^a^	3584	4388	− 804 (− 919 - − 689)
Length of hospital stay, mean (SD), days	2.6 (0.8)	2.5 (0.6)	0.1 (− 0.1–0.3)
General practitioner	634	613	28
Physiotherapist	1179	1450	− 355
Company doctor	158	182	− 31
Other primary care^b^	693	804	− 146
Care at home	886	1450	− 739
Neurosurgeon	338	298	52
Other outpatient clinic visits	734	1062	− 430
Pain medication	231	273	− 55
Emergency department^c^	505	815	− 406
Hospital admissions	1612	2437	− 1082
Additional health care costs^d^	1693	1991	− 391
Total health care costs	**12,248**	**16,055**	** − 3807 (− 6580 to − 1127)**
Absenteeism paid work^e^	8708	8818	− 110
Presenteeism paid work^e^	2434	560	1874
Absenteeism unpaid work^e^	4655	4653	2
Total productivity losses	**15,797**	**14,031**	**1767 (− 5358 to 8373)**
Total societal costs	**28,046**	**30,086**	** − 2674 (− 19,603 to 5547)**

*Values are presented as pooled estimated means in rounded Euros, unless otherwise specified

^a^Surgical costs include the price for the procedure, implants and haemostatics, and costs for days of admission to the hospital for the initial intervention

^b^Other primary care includes visits to social workers, occupational therapists, speech therapists, dieticians, alternative therapists and psychologists

^c^Emergency department includes visits to the emergency department and transportation to the hospital by ambulance

^d^Additional health care costs include day-care or admissions to a health institution other than a hospital, rehabilitation centre or psychiatric institution

^e^Absenteeism is the amount of days that a patient was absent from work times the Dutch reference wage per day. The ‘friction cost method’ was applied, assuming that after a period of 85 days of complete absence, an employee gets replaced by another worker, and productivity would be restored. Presenteeism is the loss of productivity by patients that were not able to fully fulfil their jobs. It was calculated by the proportion of productivity loss of a general working day, times the duration in days, times the average wage per day. Unpaid work is the loss of productivity during unpaid activities such as household work and volunteering. It was calculated by hours a day, times the duration in days, times the Dutch reference price for one hour of unpaid work

CI: Confidence Interval, SD: Standard deviation

### Cost-effectiveness

**Fig. 2:**
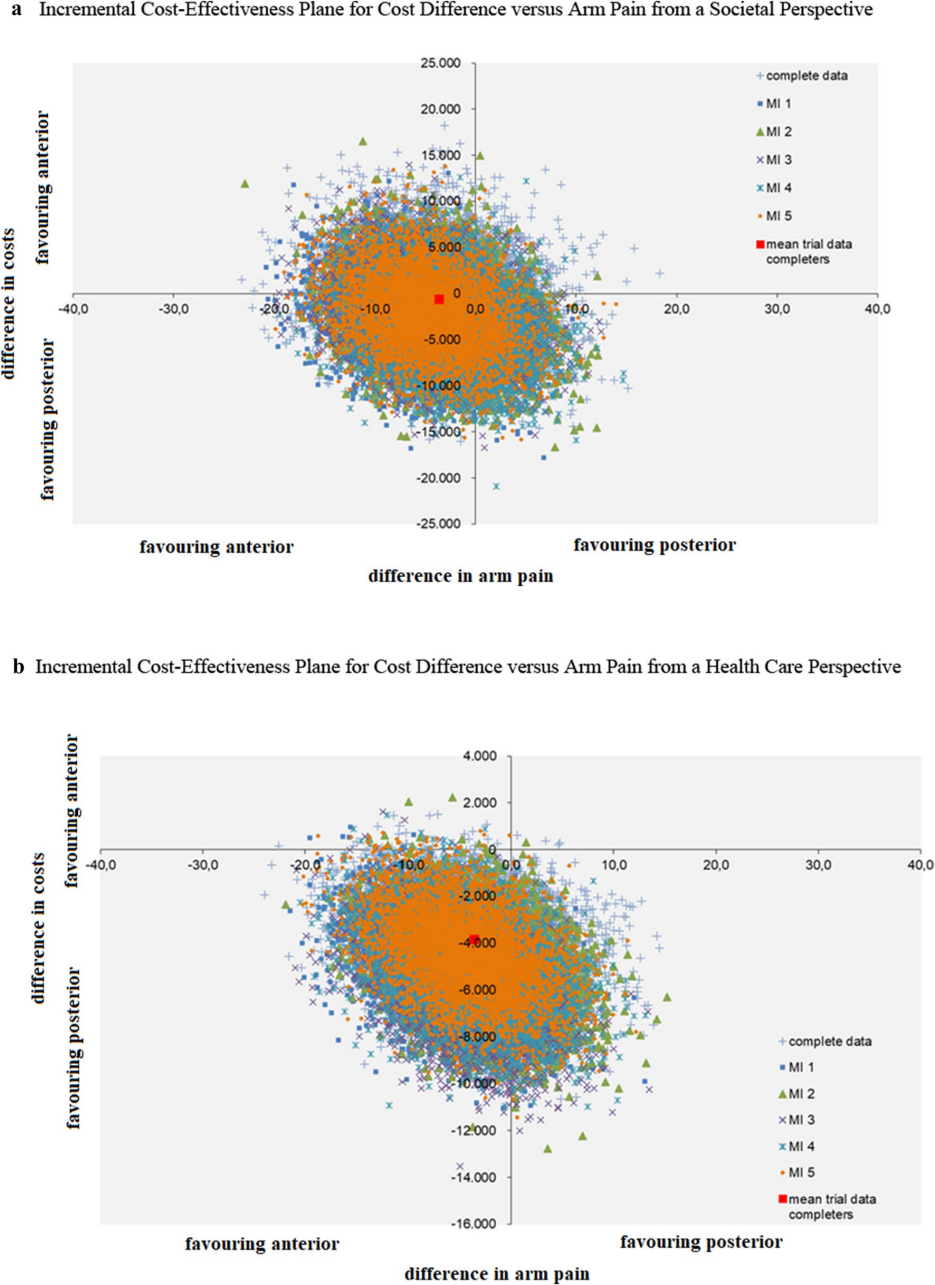

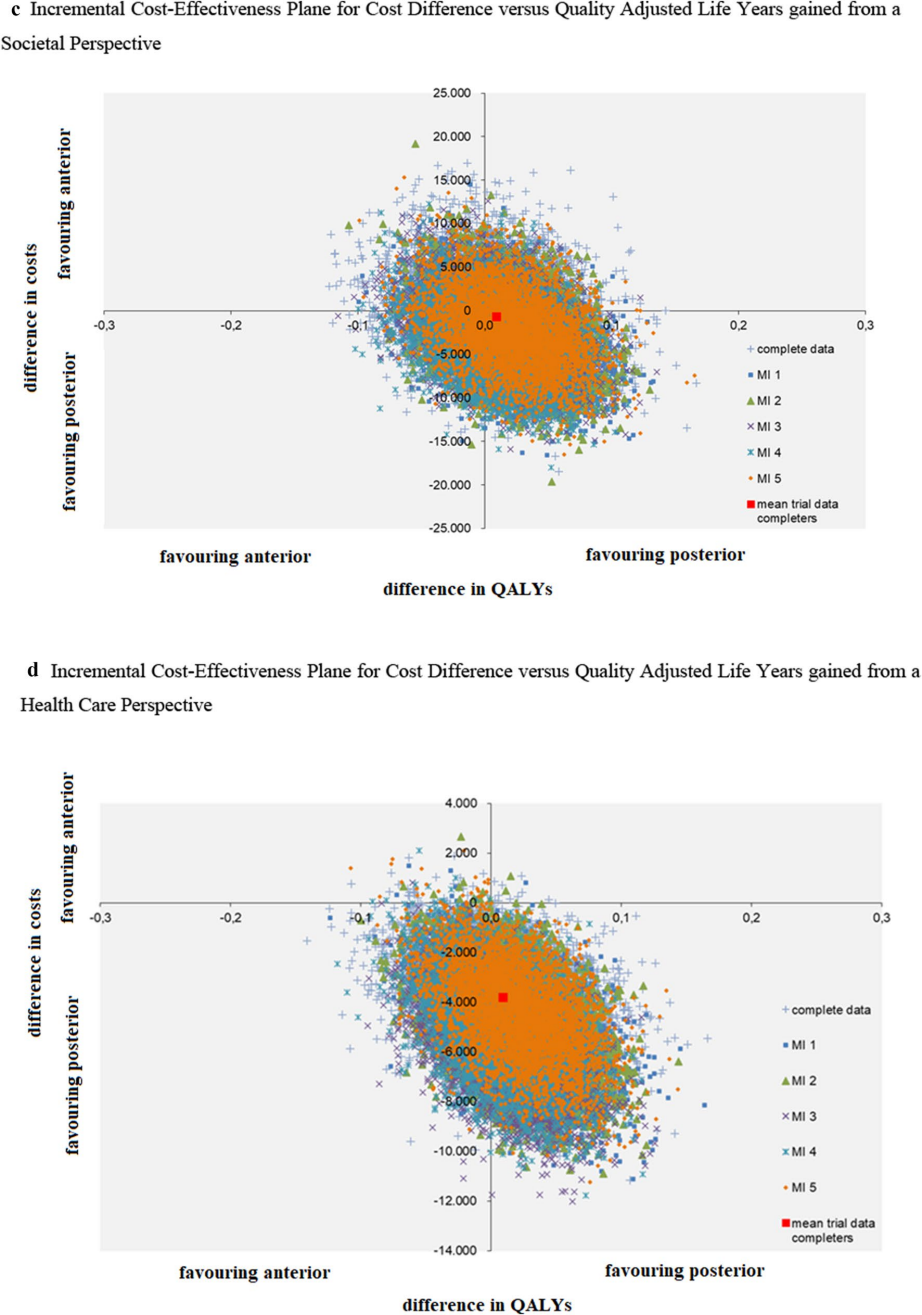
Incremental Cost-Effectiveness Planes. **a** Incremental Cost-Effectiveness Plane for Cost Difference versus Arm Pain from a Societal Perspective. **b** Incremental Cost-Effectiveness Plane for Cost Difference versus Arm Pain from a Health Care Perspective. **c** Incremental Cost-Effectiveness Plane for Cost Difference versus Quality Adjusted Life Years gained from a Societal Perspective. **d** Incremental Cost-Effectiveness Plane for Cost Difference versus Quality Adjusted Life Years gained from a Health Care Perspective. Abbreviations: Multiple imputation sets (MI)

The point estimate of cost-effectiveness, calculated with the VAS-arm from a societal perspective, was in the lower half of the ICEP, therefore it was not useful to calculate an ICER (as the interventional group was associated with lower costs and similar effectiveness compared to the control group, there would be no clinical benefits with higher costs). The ICEP depicted similar arm pain scores between groups, with high uncertainty regarding the direction of the cost difference (Fig. 2a). From a health care perspective, there was less uncertainty regarding the costs, as the various clouds of the multiple imputation sets were almost completely in the lower half, indicating that posterior surgery was associated with less healthcare costs (Fig. 2b).

### Cost-utility

**Fig. 3:**
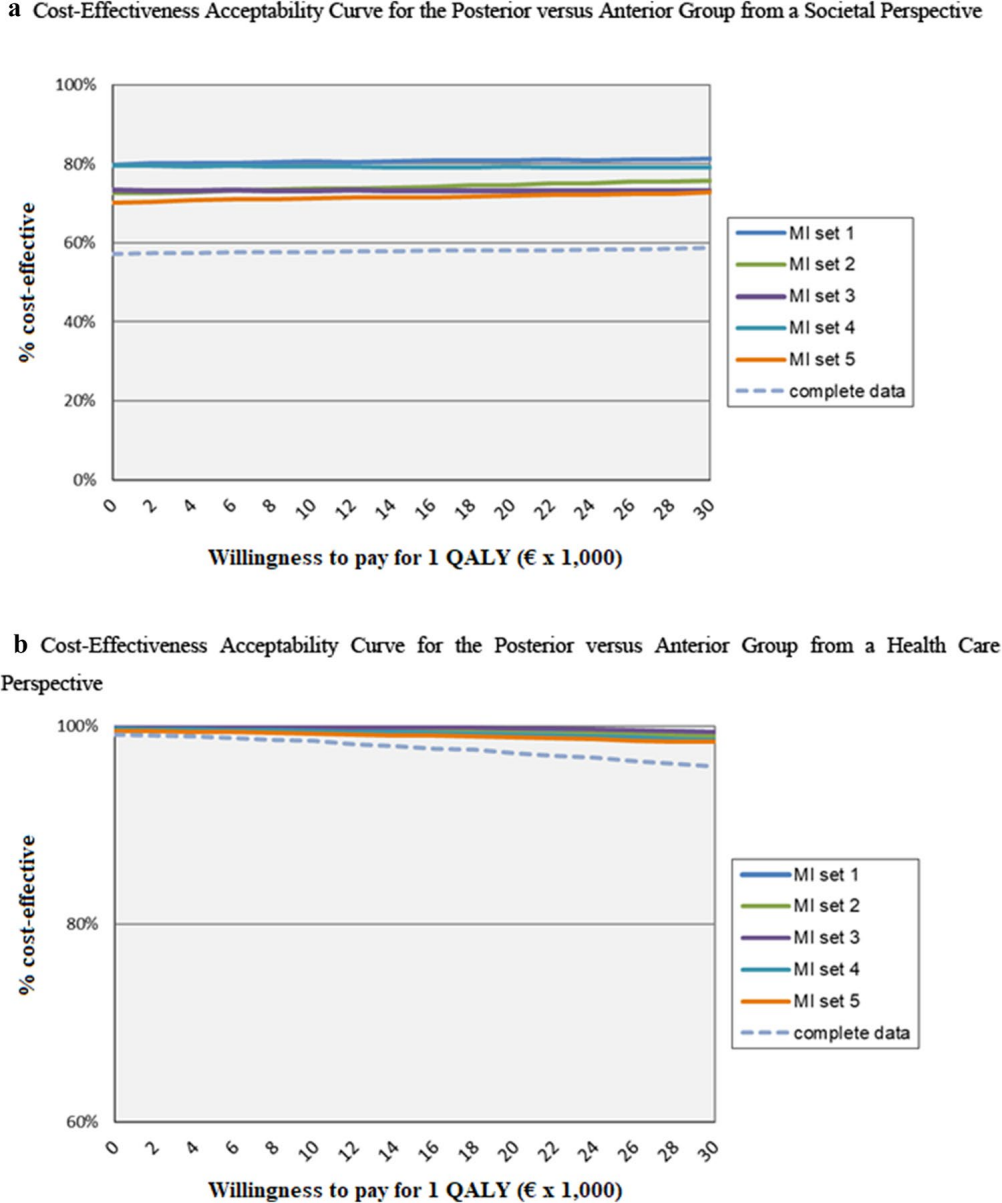
Cost-Effectiveness Acceptability Curves for the Posterior versus Anterior Group. **a** Cost-Effectiveness Acceptability Curve for the Posterior versus Anterior Group from a Societal Perspective. **b** Cost-Effectiveness Acceptability Curve for the Posterior versus Anterior Group from a Health Care Perspective. Abbreviations: Multiple imputation sets (MI), Incremental Cost-Effectiveness Ratio (ICER), Quality Adjusted Life Year (QALY)

The point estimate of cost-utility, calculated with QALYs from a societal perspective, was in the lower half of the ICEP, therefore it was also not useful to calculate an ICER. The ICEP depicted similar QALYs between groups with similar results for the cost difference as the cost-effectiveness (Fig. 2c, d). The CEACs demonstrated a high probability of posterior surgery being cost-effective. To note is that this probability includes the scenario where posterior surgery would be associated with less costs but also with less effectiveness (Fig. 3a, b). The CEAC from a societal perspective (Fig. 3a) for the group with complete data had a lower probability than the multiple imputation groups.

Figure 2a, b depict the cost difference of posterior versus anterior surgery on the vertical axis in Euros, and the difference in the Visual Analogue Scale for arm pain between groups on the horizontal axis. Figure 2a visualises the incremental cost-effectiveness plane from a societal perspective, Fig. 2b from a health care perspective.

Figure 2c, d depict the cost difference of posterior versus anterior surgery on the vertical axis in Euros, and the difference in Quality Adjusted Life Years between groups on the horizontal axis. Figure 2c visualises the incremental cost-effectiveness plane from a societal perspective, Fig. 2d from a health care perspective.

On the horizontal axis, the willingness to pay for 1 QALY gained is depicted. On the vertical axis the probability of cost-effectiveness is described. Figure 3a visualises the Cost-Effectiveness Acceptability Curve from a societal perspective, Fig. 3b from a health care perspective.

### Sensitivity analyses

ICEPs using Odom criteria and NDI as treatment effect demonstrated similar results (Supplementary Information, Figs. 1, 2, 3, 4). Analyses without medication costs demonstrated similar results, as medication costs were relatively low compared to the total costs and similar between the two groups (see Table 2).

## Discussion

The cost-effectiveness and cost-utility analyses of this multicentre randomised clinical trial comparing posterior versus anterior surgery for cervical radiculopathy demonstrated similar effectiveness with less costs associated with posterior surgery. Therefore, posterior surgery is likely to be cost-effective compared to anterior surgery. This is, to our knowledge, the first economic evaluation of these interventions based on prospectively collected data.

Three retrospective cost-effectiveness analyses in small cohorts reported favourable results for posterior surgery as well [7–9]. Although these results are in line with our study, there are some major differences compared to our study population. First, Tumiálan et al*.* described a military sample in which the anterior surgery group had strict (limiting) return to active duty regulations because of the fusion performed. As these regulations were not applicable to the posterior group, there was a large difference in productivity losses (return to full duty in 4.8 weeks (range 1–8) for the posterior group compared to 19.6 weeks (range 12–32) in the anterior group) [9]. Secondly, Mansfield et al*.* performed minimally invasive foraminotomies with same-day discharges [8]. Also, all three studies performed anterior surgery with additional plating, leading to higher differences in procedural costs between the groups. Because we did not perform anterior plating nor used neck braces, and had similar length of hospital stay in our population, it is reasonable to assume that the difference in overall costs between anterior versus posterior surgery is smaller in our study.

Furthermore, a large registry study from the United States analysed costs associated with anterior and posterior surgery and reported less direct costs for posterior surgery (mean, $16,123; ± standard deviation (SD), ± 15,393 versus $27,444 ± 17,828 in the anterior surgery group, *p* < 0.001) [10]. Together with evidence from the mentioned retrospective studies, a higher difference in surgical costs would be expected in a population where additional plating is performed, probably leading to more certainty (favouring posterior surgery) in a cost-effectiveness analysis.

Surgical costs were higher in anterior surgery which can be explained by the implants used. However, also the use of other health care resources was slightly higher in the anterior group for all subcategories. Possible explanations could be higher baseline health care resource use or a higher amount of serious adverse events during follow-up in the anterior group, although these differences were not statistically significant [3, 5]. Interestingly, comorbidities and ASA scores were slightly higher for the posterior group [3]. Unfortunately, it is not possible to correct for baseline differences in this economic analysis.

Remarkably, a higher amount of productivity loss was observed for posterior surgery in our study, specifically in presenteeism. This might be ascribed to the nature of the procedure with slightly higher short-term neck pain after surgery. Also, more recurrent radicular complaints as well as slightly more reoperations were observed after posterior surgery as previously reported, which could also have led to reduced productivity [5]. However, this did not lead to higher absenteeism in the posterior group, higher health care resource use, or statistical significant differences in patient reported outcomes. Furthermore, only a few patients reported presenteeism (Table 3, Supplementary Information). Also, baseline presenteeism was higher for posterior surgery, which might explain (parts of) the differences in productivity losses between the groups. Unfortunately, it is also not possible to correct for these baseline differences in this economic analysis. To conclude, it is uncertain whether higher productivity losses in the posterior group reflect a surgical effect. In an attempt to further study this, we will perform a secondary analysis of the FACET, where we will shed light on rehabilitation and return-to-work after posterior versus anterior surgery and explore whether differences in postoperative treatment and return-to-work trajectories exists.

### Limitations

In our analyses, the ‘friction cost method’ was applied, which assumes that a worker gets fully replaced after 85 days. Although this is recommended in the Dutch guidelines for costing research [22], a common alternative is to estimate productivity losses with the ‘human capital approach’ in which no cut-off is used for productivity losses. This could have led to higher costs for productivity losses in both groups [34].

A limitation of the performed economic evaluation is the lack of information on informal care by partners, family or friends. Furthermore, medication costs were not reported consistently by the participants, even though we used a recommended medical cost questionnaire. This could have led to an under- or over estimation of the pain medication. Sensitivity analyses, however, did not demonstrate significant changes when omitting the medication costs from the analyses.

The CEAC from a societal perspective (Fig. 3a) demonstrated a lower probability for cost-effectiveness for the complete data compared to the multiple imputation data. This could be explained by the fact that a relatively large group of patients had some missing cost data during follow-up (Fig. 1), with apparently a different cost-profile than the patients with complete data. Although the amount of patients with missing data was evenly distributed between the treatment arms, this difference in cost-profiles could have led to bias.

Furthermore, the CEACs demonstrated a high probability of posterior surgery being cost-effective at a relatively low ‘willingness-to-pay’ threshold. This can be explained by the fact that posterior surgery was associated with less costs, leading to no incremental costs for a similar clinical effectiveness. ‘Willingness-to-pay’ thresholds are different for each country and vary for severity of disease, but are in the light of the results of this study less relevant.

Cost-analyses in spine surgery are gaining popularity, although a great variety in design, reporting and outcomes exist [35, 36]. Also, the geographical distribution of studies varies substantially. As there are different financial healthcare systems and clinical practices between countries, results of studies may not be fully generalisable. Nevertheless, it is important to perform more economic evaluations in spine surgery in different geographical areas, to address the influence of regional practices and healthcare systems on the outcomes of cost evaluations. A guideline with recommendations for economic evaluations in spine surgery would aid generalisability.

In summary, posterior surgery was associated with lower costs compared to anterior surgery, and was likely to be cost-effective. As clinical outcomes were similar between groups, both approaches can be considered valid treatments for patients with cervical nerve root compression. We would like to advocate that all patients should be counselled for both approaches and a shared decision should be made with each individual patient.

## Conclusion

In patients with cervical radiculopathy due to foraminal nerve root compression, arm pain and quality of life were similar between posterior and anterior surgery. Posterior surgery was associated with lower costs, therefore posterior surgery is likely to be cost-effective compared with anterior surgery.

## Supplementary Information

Below is the link to the electronic supplementary material.

Supplementary file1 (DOCX 221 kb)

Supplementary file2 (DOCX 11210 kb)

## Data Availability

The deidentified data set, study protocol and statistical analysis plan are available on request after publication of the primary results of the study.
