# Supplementary material for: Cost-effectiveness of posterior versus anterior surgery for cervical radiculopathy: results from a multicentre randomised non-inferiority trial (FACET)
Source: Eur Spine J. 2024 Jun 7;33(8):3087–98. doi: 10.1007/s00586-024-08340-4 (PMC12611986; doi:10.1007/s00586-024-08340-4)
Supplement: Supplementary file 1 — Supplementary file1 (DOCX 221 kb) [file 586_2024_8340_MOESM1_ESM.docx]

# **Final Statistical Analysis Plan**

Cost-effectiveness of posterior versus anterior surgery

for cervical radiculopathy:

results from a multicentre randomised non-inferiority trial (FACET)

Abbreviations:

| ACDF | Anterior Cervical Discectomy with Fusion |
| --- | --- |
| CEAC | Cost-Effectiveness Acceptability Curve |
| DBC | Diagnosis Treatment Combination |
| DRG | Diagnosis Related Groups |
| EQ-5D-5L | EuroQol 5-Dimensions 5-Level |
| FACET | Foraminotomy ACDF Cost-Effectiveness Trial |
| FOR | Posterior Cervical Foraminotomy |
| ICER | Incremental Cost-Effectiveness Ratio |
| ICEP | Incremental Cost-Effectiveness Plane |
| iMCQ | Institute for Medical Technology Assessment Medical Consumption Questionnaire |
| iPCQ | Institute for Medical Technology Assessment Productivity Cost Questionnaire |
| NDI | Neck Disability Index |
| SAP | Statistical Analysis Plan |
| VAS | Visual Analogue Scale |

## Research aims and hypotheses*:

The overall hypotheses of the FACET were:

Primary hypotheses:

1. The effectiveness of the FOR technique is non-inferior compared with the ACDF technique
2. **The FOR technique is cost-effective compared with the ACDF technique**

Secondary hypotheses:

1. The FOR technique will have a lower complication rate compared with the ACDF technique.
2. The FOR technique will have lower direct and indirect costs compared with the ACDF technique.
3. The FOR technique is associated with more neck pain in the first 30 days after the surgical procedure.

In this SAP, details regarding the second primary hypothesis (FOR is cost-effective compared to ACDF) are described.

## Study design and treatment allocation*:

The FACET is a prospective, multicentre, investigator-blinded randomised controlled trial with a follow-up of 2 years. Both the FOR (experimental group) and the ACDF (active control) are established surgical techniques. The randomisation was performed using an independent institute web-based block randomisation design, stratified by centre. Blinding of the participant or the surgeon is not feasible. The data analysis was performed with blinded data.

## Sample size*

A sample size of 308 patients was calculated that would give the trial 80% power to rule out a between-group difference in the success rate with an alpha of 0.05, and a drop-out ratio of 10%, and non-inferiority margin of 10%. During the trial, a lower inclusion rate was observed and several measures to improve inclusion were taken. This included frequent newsletters, defined instructions for surgeons to include participants and the enrollment of additional medical centres. Nonetheless, a delay in inclusion could not be fully alleviated. When also the COVID-pandemic started, with an extreme reduction of non-emergent health care, an interim analysis and post-hoc power calculation were performed by a statistician who was not involved in the study design.

*According to original and published protocol: Broekema, A. E. H., Kuijlen, J. M. A., Lesman-Leegte, G. A. T., Bartels, R. H. M. A., Van Asselt, A. D. I., Vroomen, et al. (2017). Study protocol for a randomised controlled multicentre study: The Foraminotomy ACDF Cost-Effectiveness Trial (FACET) in patients with cervical radiculopathy. *BMJ Open,* 7(1). https://doi.org/10.1136/bmjopen-2016-012829

The interim-analysis included participants that completed the 1-year follow-up and those that completed the 2-year follow-up at that time (the 2-year follow-up is still ongoing, thus not completed for all patients yet).

The analysis included the primary outcome on which the sample size was based on; a successful Odom score (‘excellent’ or ‘good’). Besides a complete case analysis, additional sensitivity analyses were performed; a scenario with full cases in which all missing cases were considered to have an unsuccessful outcome; a scenario with predefined sample sizes (140 participants per group) with all remaining missing data coded as unsuccessful; and a scenario in which additional participants in the ACDF group were coded as unsuccessful (stacking the Odds against FOR) until the non-inferiority margin was reached (Figure 1). All scenario’s indicated non-inferiority of FOR with a confidence interval within the predefined delta of 0.1. Therefore, we could conclude that it was safe to end the inclusion at 86% of the predefined sample size.


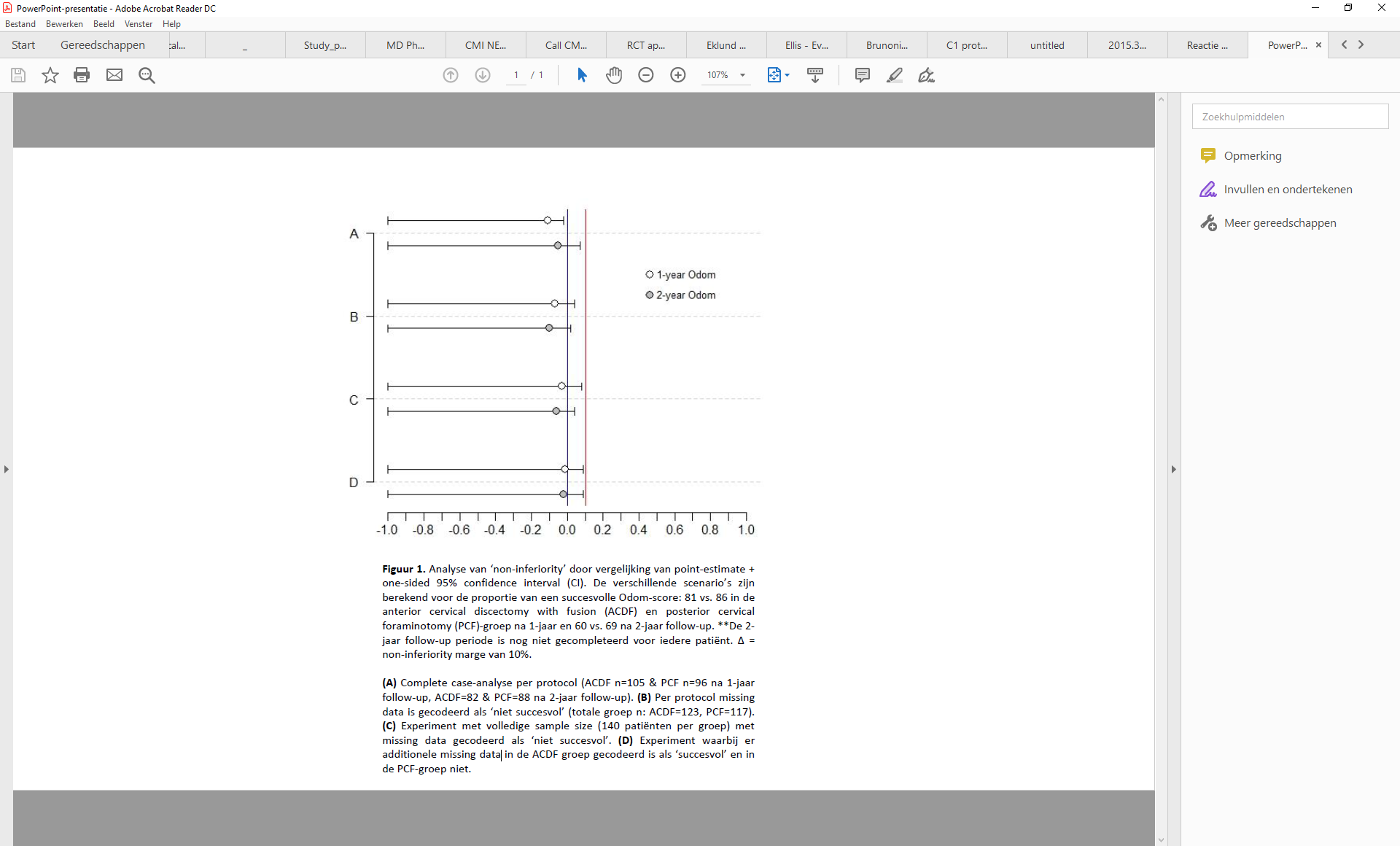


**Figure 1. Interim-analyses of non-inferiority.**

Point estimate + one-sided 95% confidence interval (CI) are depicted. Different scenarios were calculated for the proportion of a successful primary outcome (Odom score): 86 versus 81 in the FOR and ACDF group after 1-year of follow-up, and 69 versus 60 after 2-years of follow-up, respectively. **the 2-year follow-up is still ongoing and thus not completed for all patients. The red line depicts the non-inferiority margin of 10%.

**(A)** Intention-to-treat analysis with complete cases after 1- and 2-years of follow-up. **(B)** Intention-to-treat analysis with full cases (FOR 119 and ACDF 124), with missing data coded as unsuccessful. **(C)** Analysis with predefined sample sizes (140 patients per group) with missing data coded as unsuccessful **(D)** Scenario where additional patients in the ACDF group were coded as having a successful outcome, until the non-inferiority margin was reached (stacking the odds against FOR).

## Data collection methods*:

Preoperative history is obtained from standard care procedures and includes length, weight, number of months/years of neck and arm pain, signs and symptoms, other significant illnesses, pain medication (use of non-steroidal anti-inflammatory drugs) and smoking history. Information about the operative procedure will be obtained from the medical record of the participant and will include date and type of procedure, which level was operated, use of implants and occurrence of complications during the operative procedure. At baseline (i.e. at enrolment, before surgical procedure), participants will fill out web-based questionnaires. These questionnaires take ~30 min to fill in. The participant will visit the outpatient clinic 6 weeks after the surgical procedure, in line with standard care. At 6, 26, 52, 78 and 104 weeks after surgery, the participant fills out the same abovementioned questionnaires. An independent interviewer will contact the participants by telephone at all follow-up moments to assess the Odom criteria. During the complete period of the study, all adverse events will be reported. Adverse events are defined as undesirable experience occurring to a participant during the study, whether or not considered related to intervention. The definition of serious adverse events is in line with the guidelines of the International Council on Harmonization of Technical Requirements for Registration of Pharmaceuticals for Human Use.^1^

The costs of healthcare resource use were derived from the Institute for Medical Technology Assessment Medical Consumption Questionnaire (iMCQ) by multiplying visits to caregivers, procedures performed, clinical admissions, medication use, and other healthcare consumption by standard unit prices as recommended in the Dutch Costing Manual^2^. If necessary, prices were converted to the 2020 price level using the price index provided by the Dutch Central Bureau of Statistics^3^. Pain medication costs were based on generic prices from the Dutch “Pharmacotherapeutic Compass”^4^. Centre-specific surgical costs were drawn from the Dutch ‘diagnosis treatment combination’ (DBC) system, which is similar to the ‘diagnosis related groups’ (DRG) billing system used in many European countries, and included prices for implants, microscopes and hemostatics^5^. Admission days and visits to the outpatient clinic, related to the primary intervention, were separately calculated (in contrast to using the cost price for the DBC, which already includes the average costs for hospital admission and outpatient clinic visits).

Productivity losses were assessed with the validated Institute for Medical Technology Assessment Productivity Cost Questionnaire (iPCQ)^6^. For productivity losses in paid work, absenteeism (complete absence from work) as well as presenteeism (productivity loss during work) were distinguished. Absenteeism was calculated by the amount of days that a patient was absent from work times the Dutch reference wage per day. The ‘friction cost method’ was applied, assuming that after a period of 85 days of complete absence, an employee gets replaced by another worker, and productivity would be restored^7,8^.

*According to original and published protocol: Broekema, A. E. H., Kuijlen, J. M. A., Lesman-Leegte, G. A. T., Bartels, R. H. M. A., Van Asselt, A. D. I., Vroomen, et al. (2017). Study protocol for a randomised controlled multicentre study: The Foraminotomy ACDF Cost-Effectiveness Trial (FACET) in patients with cervical radiculopathy. *BMJ Open,* 7(1). https://doi.org/10.1136/bmjopen-2016-012829

Presenteeism was calculated by the proportion of productivity loss of a general working day, times the duration in days, times the average wage per day. Unpaid work is the loss of productivity during unpaid activities such as household work and volunteering. It was calculated by hours a day, times the duration in days, times the Dutch reference price for one hour of unpaid work.

The recall period, meaning the time period that is questioned, was three months for the iMCQ and iPCQ (with exception for the questionnaire 6 weeks after surgery using a recall period of 6 weeks). Therefore, values of the measurements at 52, 78 and 104 weeks were doubled, with exception of hospital treatments and admissions. All costs were expressed in U.S. Dollars and were converted from Euros using the 2020 purchasing power parity conversion rate from the Organisation for Economic Co-operation and Development^9^.

## Outcome measures:

Two primary outcomes were defined:

1. Cost-effectiveness from a societal perspective, based on VAS arm pain.
2. Cost-utility from a societal perspective, based on QALYs gained.

Cost-effectiveness (based on VAS-arm) and cost-utility (based on QALYs) from a health care perspective, meaning all costs related to health care resource use without costs related to productivity losses, were considered secondary outcomes.

## Statistical methods: primary study parameters:

The societal perspective includes all costs related to health care resource use and productivity losses. A time horizon of 2 years was used. According to Dutch pharmaco-economic guidelines^7^, discounting was applied for costs (4%) and effects (1.5%) in the second year to unify costs and effects occurring in different years.

Incremental cost-effectiveness ratios (ICERs) were calculated for FOR compared to ACDF. An ICER is the difference in costs between two interventions, divided by the difference in their (clinical) effect. First, incremental costs for relief in arm pain were calculated, based on the area under the curve for VAS arm pain (0-100 millimeter).

Secondly, incremental costs per QALY gained were calculated using the EQ-5D-5L^10^. The EQ-5D-5L scores were converted into utility scores (-0.446 – 1) based on the Dutch tariffs^11^. QALYs were calculated by multiplying utility scores by time spent in each health state.

## Statistical methods: secondary study parameters:

Secondary outcomes, being cost-effectiveness (VAS arm pain) and cost-utility (QALYs gained) from a health care perspective, were calculated with the same method as the primary outcomes. The ‘health care perspective’ included all costs related to health care resource use, without costs related to productivity losses.

## Missing data

Missing data in costs and patient reported outcomes were handled by performing multiple imputation with five imputation sets. For each imputation set, cost-effectiveness was bootstrapped separately with 5,000 resamples and data were reported as pooled means with bootstrapped 95% confidence intervals (CI). Results of all analyses were presented in incremental cost-effectiveness planes (ICEPs) and cost-effectiveness acceptability curves (CEACs). CEACs visualise the probability that an intervention is cost-effective, given a certain societal willingness to pay for a millimeter decrease in arm pain or for one QALY gained.

Sensitivity analyses were performed by comparing the results of the primary analyses with similar analyses in complete cases only. Furthermore, cost-effectiveness analysis with the VAS arm pain as effect was compared to analyses using proportion of success based on the Odom criteria (4-point rating scale, ‘excellent’ and ‘good’ considered as successful)^12,13^ as well as the Neck Disability Index (NDI)^13,14^ as treatment effect. For the dichotomised Odom criteria, available data at 2 year follow-up was used, as it was not possible to perform reliable multiple imputations. SPSS software (version 28.0, IBM Corp., Armonk, NY) was used for data analysis and Microsoft Excel 365 for bootstrapping simulation.

## References:

1. International Conference on Harmonisation of Technical Requirements for Registration of Pharmaceuticals for Human Use. *ICH harmonized tripartite guideline: Clinical safety data management: Definitions and standards for expedited reporting E2A.* Step 4 version ed. October.1994.http://www.ich.org/fileadmin/Public_Web_Site/ICH_Products/Guidelines/Efficiacy/E2A/Step4/E2A_Guideline.pdf Accessed on 4^th^ of March, 2023.
2. Swan Tan S, Bouwmans-Frijters CAM, Hakkaart-van Roijen L, Kanters T, Ts for economic evaluations in healthcare. Handleiding voor kostenonderzoek: methoden en referentieprijzen voor economische evaluaties in de gezondheidszorg. *Tijdschrift voor gezondheidswetenschappen*. 2012;90(6). doi:10.1007/s12508-012-0128-3
3. Central Bureau of Statistics. Consumer Price Index. https://opendata.cbs.nl/statline/#/CBS/nl/dataset/70936ned/table.
4. Cv Zorgverzekeringen IB. Farmacotherapeutisch Kompas. https://www.farmacotherapeutischkompas.nl/.
5. Oostenbrink JB, Rutten FFH. Cost assessment and price setting of inpatient care in the Netherlands. The DBC case-mix system. *Health Care Manag Sci*. 2006;9(3). doi:10.1007/s10729-006-9096-y
6. Bouwmans C, Krol M, Severens H, Koopmanschap M, Brouwer W, Roijen LH van. The iMTA Productivity Cost Questionnaire: A Standardized Instrument for Measuring and Valuing Health-Related Productivity Losses. *Value in Health*. 2015;18(6). doi:10.1016/j.jval.2015.05.009
7. Hakkaart-van Roijen L, van der Linden N, Bouwmans CAM, Kanters T, Tan SS. *Kostenhandleiding: Methodologie van Kostenonderzoek En Referentieprijzen Voor Economische Evaluaties in de Gezondheidszorg.* Erasmus University Rotterdam: Institute for Medical Technology Assessment; 2015.
8. Koopmanschap MA, Rutten FFH, van Ineveld BM, van Roijen L. The friction cost method for measuring indirect costs of disease. *J Health Econ*. 1995;14(2). doi:10.1016/0167-6296(94)00044-5
9. Organisation for Economic Co-operation and Development. Purchasing power parities (PPP).
10. Janssen MF, Pickard AS, Golicki D, et al. Measurement properties of the EQ-5D-5L compared to the EQ-5D-3L across eight patient groups: A multi-country study. *Quality of Life Research*. Published online 2013. doi:10.1007/s11136-012-0322-4
11. Versteegh M, M. Vermeulen K, M. A. A. Evers S, de Wit GA, Prenger R, A. Stolk E. Dutch Tariff for the Five-Level Version of EQ-5D. *Value in Health*. 2016;19(4). doi:10.1016/j.jval.2016.01.003
12. Odom GL, Finney W, Woodhall B. Cervical disk lesions. *J Am Med Assoc*. 1958;166(1). doi:10.1001/jama.1958.02990010025006
13. Broekema AEH, Molenberg R, Kuijlen JMA, Groen RJM, Reneman MF, Soer R. The Odom Criteria: Validated at Last: A Clinimetric Evaluation in Cervical Spine  Surgery. *J Bone Joint Surg Am*. 2019;101(14):1301-1308. doi:10.2106/JBJS.18.00370
14. Vernon H, Mior S. The neck disability index: A study of reliability and validity. *J Manipulative Physiol Ther*. Published online 1991.
15. Jorritsma W, de Vries GE, Dijkstra PU, Geertzen JHB, Reneman MF. Neck Pain and Disability Scale and Neck Disability Index: Validity of Dutch language versions. *European Spine Journal*. Published online 2012. doi:10.1007/s00586-011-1920-5

## Summary of changes in Statistical Analysis Plan

|  | **Original protocol** | **Published protocol and present report** | **Summary of changes** |
| --- | --- | --- | --- |
| **Primary hypothesis*** | The FOR technique is cost-effective compared with the ACDF technique | The FOR technique is cost-effective compared with the ACDF technique | No changes |
| **Study design and treatment allocation*** |  |  |  |
| Non-inferiority margin | A non-inferiority trial design is chosen to show whether FOR has at least as much efficacy as the ACDF technique or is worse by an amount <10% with regard to the primary outcome parameters. | A non-inferiority trial design is chosen to show whether FOR has at least as much efficacy as the ACDF technique or is worse by an amount <10% with regard to the primary outcome parameters. | No changes |
| Sample size | A sample size of 308 patients was calculated that would give the trial 80% power to rule out a between-group difference in the success rate with an alpha of 0.05, a drop-out ratio of 10%, and non-inferiority margin of 10%. | The interim-analysis included participants that completed the 1-year follow-up and participants that included the 2-year follow-up at that time (the 2-year follow-up is still ongoing). The analysis was based on the prespecified primary outcome a successful Odom score (‘excellent’ or ‘good’), on which the initial sample size was also based. Besides a ‘complete case’ analysis, additional sensitivity analyses were performed. All scenario’s indicated non-inferiority of posterior cervical foraminotomy with a confidence interval within the predefined delta of 0.1. Therefore, we could conclude that it was safe to end the inclusion at 86% of the predefined sample size (265 patients). | 265 patients were included in the trial (86% of the predefined sample size) and 243 patients received the final treatment. |
| **Outcome measures** |  |  |  |
| Primary outcome | There will be two separate outcome measures for the cost- effectiveness analysis, resulting in two incremental cost-effectiveness ratios (ICERs) for FOR as compared to ACDF, the first is incremental costs per extra percentage of patients with arm pain relief, the second is incremental costs per Quality Adjusted Life Year (QALY) gained. The analysis will be performed taking a societal perspective. The time horizon will be equivalent to the full follow-up of the clinical study, which is 24 months. | The cost-effectiveness analysis will be performed  alongside the clinical trial to assess the cost-effectiveness  of FOR versus ACDF. There will be two separate  outcome measures for the cost-effectiveness analysis,  resulting in two incremental cost-effectiveness ratios for  FOR as compared with ACDF. The first is incremental costs per extra percentage of patients with arm pain relief, while the second is incremental costs per QALY gained. EQ-5D-5L scores will be converted into health  state utilities using the Dutch value set.33 These health state utilities range between −0.446 and 1, with a higher utility indicating a better health-related QoL. The utilities  will be multiplied by follow-up time spent in that  particular health state (area under the curve) to eventually convert into QALYs. The analysis will be performed taking a societal perspective.  The time horizon will be equivalent to the full  follow-up of the clinical study, which is 24 months. | No changes |
| Secondary outcomes | *Secondary outcomes for the cost-effectiveness analysis were not defined in the original protocol.* | Cost-effectiveness (based on VAS-arm) and cost-utility (based on QALYs) from a health care perspective, meaning all costs related to health care resource use without costs related to productivity losses, were considered secondary outcomes. | Cost-effectiveness and cost-utility from a health care perspective were added as secondary outcome to provide relevant information for health care professionals. |
| **Statistical analysis of primary outcome** | The first outcome for the cost-effectiveness analysis is the percentage of patients with arm pain relief, which will be measured and analysed by the VAS, Odom's criteria, neurological testing and the NDI for the extent of neck pain. The second outcome is QALYs, which are assessed by means of the EQ-5D-5L questionnaire. EQ-5D-5L scores will be converted into health state utilities, ranging between 0 and 1, with a higher utility indicating a better health-related Quality of Life (QoL). These utilities will be multiplied with follow-up time spent in that particular health state (area under the curve) to eventually convert into QALYs. | There will be two separate  outcome measures for the cost-effectiveness analysis,  resulting in two incremental cost-effectiveness ratios for  FOR as compared with ACDF. The first is incremental costs per extra percentage of patients with arm pain relief, while the second is incremental costs per QALY gained. Regarding patient reported outcomes, the area under the curve was calculated using all time points.  Results of all analyses were presented in incremental cost-effectiveness planes (ICEPs) and cost-effectiveness acceptability curves (CEACs). CEACs visualise the probability that an intervention is cost-effective, given a certain societal willingness to pay for a millimeter decrease in arm pain or for one QALY gained. | Arm pain relief was clarified in the final report as the area under the curve using the VAS arm pain score. Cost-effectiveness with the other patient reported outcome measures mentioned in the original report (Odom criteria and NDI) were used as treatment effect for sensitivity analyses. |
| **Statistical analysis of secondary outcome** | *Secondary outcomes for the cost-effectiveness analysis were not defined in the original protocol.* | Secondary outcomes were analysed with the same method as the primary outcomes. |  |
| **Missing data** | For the purpose of a supportive sensitivity analysis, multiple imputation procedures will be applied. | Missing data in costs and patient reported outcomes were handled by performing multiple imputation with five imputation sets. For each imputation set, cost-effectiveness was bootstrapped separately with 5,000 resamples and data were reported as pooled means with bootstrapped 95% confidence intervals (CI).  Sensitivity analyses were performed by comparing the results of the primary analyses with similar analyses in complete cases only. Furthermore, cost-effectiveness analysis with the VAS arm pain as effect was compared to analyses using proportion of success based on the Odom criteria (4-point rating scale, ‘excellent’ and ‘good’ considered as successful) as well as the Neck Disability Index (NDI) as treatment effect. | Methods for handling missing data and performing sensitivity analyses were described in more detail in the final report. |
| **Other** | British English is used in our original statistical analysis plan (according to the published protocol). | American English is used in our final statistical analysis plan. | Change of British English to American English. |
