# Supplementary material for: Cost-effectiveness of posterior versus anterior surgery for cervical radiculopathy: results from a multicentre randomised non-inferiority trial (FACET)
Source: Eur Spine J. 2024 Jun 7;33(8):3087–98. doi: 10.1007/s00586-024-08340-4 (PMC12611986; doi:10.1007/s00586-024-08340-4)
Supplement: Supplementary file 2 — Supplementary file2 (DOCX 11210 kb) [file 586_2024_8340_MOESM2_ESM.docx]

**Supplementary Information**

**Cost-effectiveness of posterior versus anterior surgery for cervical radiculopathy: results from a multicentre randomised non-inferiority trial (FACET).**

**
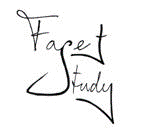
**

FACET
Foraminotomy ACDF Cost-Effectiveness Trial

**TABLE OF CONTENTS**

**Page**

**List of FACET investigators** 3

**Original study protocol** 7

**Supplementary Tables**

Table 1 Time points and calculations for iMCQ and iPCQ 29

Table 2 Baseline healthcare resource use and productivity losses 30

Table 3. Cost differentiation with number of patients reporting costs 31

**Supplementary Figures**

Figure 1 Incremental Cost-Effectiveness Plane comparing Cost Difference and 33

Neck Disability Index from a Societal Perspective

Figure 2 Incremental Cost-Effectiveness Plane comparing Cost Difference and 34

Neck Disability Index from a Healthcare Perspective

Figure 3 Incremental Cost-Effectiveness Plane comparing Cost Difference and 35

Odom Criteria from a Societal Perspective

Figure 4 Incremental Cost-Effectiveness Plane comparing Cost Difference and 36

Odom Criteria from a Healthcare Perspective

**List of FACET investigators**

A.E.H. Broekema, MD

Department of Neurosurgery

University of Groningen, University Medical Centre Groningen

Groningen, The Netherlands

N.F. Simões de Souza, BSc

Department of Neurosurgery

University of Groningen, University Medical Centre Groningen

Groningen, The Netherlands

R. Soer, PhD

Groningen Pain Centre

University of Groningen, University Medical Centre Groningen

Saxion University of Applied Sciences Enschede

Groningen, The Netherlands

M.F. Reneman, PhD

Department of Rehabilitation

University of Groningen, University Medical Centre Groningen

Groningen, The Netherlands

J.M.A. Kuijlen, MD PhD

Department of Neurosurgery

and Groningen Spine Centre

University of Groningen, University Medical Centre Groningen

Groningen, The Netherlands

R.J.M. Groen, MD PhD

Department of Neurosurgery

University of Groningen, University Medical Centre Groningen

Groningen, The Netherlands

J.M.C. van Dijk, MD PhD

Department of Neurosurgery

University of Groningen, University Medical Centre Groningen

Groningen, The Netherlands

J. Koopmans, MD PhD

Department of Neurosurgery

Martini Hospital Groningen

Groningen, The Netherlands

P. Plas

Department of Neurosurgery

Martini Hospital Groningen

Groningen, The Netherlands

R.H.M.A. Bartels, MD PhD

Department of Neurosurgery

Radboud University Medical Centre Nijmegen and Canisius Wilhelmina Hospital Nijmegen

Nijmegen, The Netherlands

S. van Kuijk

Department of Neurosurgery

Canisius Wilhelmina Hospital Nijmegen

The Netherlands

H. van Santbrink, MD PhD

Department of Neurosurgery

Zuyderland Medical Centre Heerlen

Heerlen, The Netherlands

K. Rijkers, MD PhD

Department of Neurosurgery

Zuyderland Medical Centre Heerlen

Heerlen, The Netherlands

R. Giepmans

Department of Neurosurgery

Zuyderland Medical Centre Heerlen

Heerlen, The Netherlands

M. Arts, MD PhD

Department of Neurosurgery

Medical Centre Haaglanden

Den Haag, The Netherlands

R. van Zijl

Department of Neurosurgery

Medical Centre Haaglanden

Den Haag, The Netherlands

I. van Beelen

Department of Neurosurgery

Medical Centre Haaglanden

Den Haag, The Netherlands

N. Hoess, MD PhD

Department of Neurosurgery

Medisch Spectrum Twente

The Netherlands

T. Bruggink

Department of Neurosurgery

Medisch Spectrum Twente

The Netherlands

N. van der Gaag, MD PhD

Department of Neurosurgery

Hagaziekenhuis

Den Haag, The Netherlands

B. Burhani, MD PhD

Department of Neurosurgery

Elisabeth Tweesteden Ziekenhuis

Tilburg, The Netherlands

P. van Limpt-Vriens

Department of Neurosurgery

Elisabeth Tweesteden Ziekenhuis

Tilburg, The Netherlands

M.P.H. Verhagen, MD

Department of Neurosurgery

Noordwest Ziekenhuisgroep

Alkmaar, The Netherlands

A.D.I. van Asselt, PhD

Department of Epidemiology

and Department of Pharmacy

University of Groningen, University Medical Centre Groningen

Groningen, The Netherlands

K. Tamasi, PhD

Department of Epidemiology and Neurosurgery,

University of Groningen, University Medical Centre Groningen

Groningen, The Netherlands

D.H. Steenks

Department of Neurosurgery

University of Groningen, University Medical Centre Groningen

Groningen, The Netherlands

G.A.T. Lesman-Leegte, RN PhD

Department of Epidemiology

University of Groningen, University Medical Centre Groningen

The Netherlands

**ORIGINAL RESEARCH PROTOCOL**

(as submitted to and approved by the research ethical board of the University Medical Centre Groningen, the Netherlands)

**Cost-effectiveness of posterior cervical foraminotomy(FOR) versus anterior cervical discotomy with fusion (ACDF) for cervical soft/osteophytic disc disease.**

**A randomized controlled multiCentre study.**

**FACET study**

**(F**oraminotomy **A**cdf **C**ost-**E**ffectiveness **T**rial)

**Cost-effectiveness of posterior cervical foraminotomy (FOR) versus anterior cervical discotomy with fusion (ACDF) for cervical soft/osteophytic disc disease. A randomized controlled multiCentre study (FACET study).**

| **Protocol ID** | **UMCG register: 201500354** |
| --- | --- |
| **Short title** | **F**oraminotomy **A**cdf **C**ost-**E**ffectiveness **T**rial  **(FACET study)** |
| **Version** | **1.0 final** |
| **Date** | **25-08-2015** |
| **Coordinating investigator/project leader** | **Dr. J.M.A. Kuijlen, MD, PhD**  Neurochirurg  *T:* 050-3612837 *\| F:* 050-3611715 *\| E:* [j.m.a.kuijlen@umcg.nl](mailto:j.m.a.kuijlen@umcg.nl) Universitair Medisch Centrum Groningen  Neurochirurgie Postbus 30001  9700 RB GRONINGEN |
| **Principal investigator** | **Prof. dr. R.J.M. Groen MD PhD**  Afdelingshoofd Neurochirurgie  *T:* 050-3612837 *\| F: 050-3611715 \| E:* [r.j.m.groen@umcg.nl](mailto:r.j.m.groen@umcg.nl) Universitair Medisch Centrum Groningen  Neurochirurgie Postbus 30001  9700 RB GRONINGEN |
| **Other participating centres:** | **Martini Ziekenhuis Groningen Medisch Spectrum Twente, Enschede Atrium/Orbis Ziekenhuis, Heerlen**  **St. Radboud Universitair Medischl Centrum, Nijmegen**  **Medisch Centrum Haaglanden, Den Haag** |
| **Sponsor** | **UMCG (Administrative responsibility)** Universitair Medisch Centrum Groningen Postbus 30001  9700 RB GRONINGEN |
| **Subsidising party** | **ZONMW** |
| **Independent expert (s)** | E.W. Hoving, MD, PhD Neurochirurg  *T:* 050-3612837 *\| F:* 050-3611715 *\| E:* [*e.w.hoving@umcg.nl*](mailto:e.w.hoving@umcg.nl)  Universitair Medisch Centrum Groningen Neurochirurgie  Postbus 30001  9700 RB GRONINGEN |
| **Study coordination support** | Trial Coordination Centre,  Universitair Medisch Centrum Groningen |

**LIST OF ABBREVIATIONS AND RELEVANT DEFINITIONS**

| **ABR** | **ABR form, General Assessment and Registration form, is the application form that is required for submission to the accredited Ethics Committee (In Dutch, ABR = Algemene**  **Beoordeling en Registratie)** |
| --- | --- |
| **ACDF** | **Anterior Cervical Discotomy with fusion** |
| **AE** | **Adverse Event** |
| **ANCOVA** | **Analysis of Covariance** |
| **ASA** | **The American Society of Anesthesiologists** |
| **BIA** | **Budget Impact Analysis** |
| **BKZ** | **Budgettair Kader Zorg** |
| **CCMO** | **Central Committee on Research Involving Human Subjects; in Dutch: Centrale**  **Commissie Mensgebonden Onderzoek** |
| **CRF** | **Case Report Form** |
| **CRS** | **Cervical Radicular Syndrome** |
| **CT** | **Computed Tomography** |
| **DSMB** | **Data Safety Monitoring Board** |
| **EQ-5D** | **EuroQol-5D** |
| **FACET** | **Foraminotomy Acdf Cost-Effectiveness Trial** |
| **FOR** | **Posterior cervical Foraminotomy** |
| **GCP** | **Good Clinical Practice** |
| **GP** | **General Practitioner** |
| **ICER** | **Incremental Cost-effectiveness ratio** |
| **ISPOR** | **International Society for Pharmacoeconomics and Outcomes Research** |
| **iMCQ** | **Institute for Medical Technology Assessment-Medical Costs Questionnaire** |
| **iPCQ** | **Institute for Medical Technology Assessment-Productivity Costs Questionnaire** |
| **METC** | **Medical Research Ethics Committee (MREC); in Dutch: Medisch Ethische Toetsing**  **commissie (METc)** |
| **MRI** | **Magnetic Resonance Imaging** |
| **NDI** | **Neck Disability Index** |
| **NSAID** | **Non-Steroidal Anti-Inflammatory Drugs** |
| **PI** | **Principle Investigator** |
| **QALY** | **Quality Adjusted Life Years** |
| **RCT** | **Randomized Clinical Trial** |
| **(S)AE** | **(Serious) Adverse Event** |
| **Sponsor** | **The sponsor is the party that commissions the organisation or performance of the**  **research, for example a pharmaceutical company, academic hospital, scientific organisation or investigator. A party that provides funding for a study but does not** |

|  | **commission it is not regarded as the sponsor, but referred to as a subsidising party.** |
| --- | --- |
| **SUSAR** | **Suspected Unexpected Serious Adverse Reaction** |
| **TCC** | **Trial Coordination Centre** |
| **UMCG** | **University Medical Centre Groningen** |
| **VAS** | **Visual Analogue Scale** |
| **WAI** | **Work Ability index** |
| **Wbp** | **Personal Data Protection Act (in Dutch: Wet Bescherming Persoonsgegevens)** |
| **WMO** | **Medical Research Involving Human Subjects Act (in Dutch: Wet Medisch-**  **wetenschappelijk Onderzoek met Mensen)** |

**SUMMARY**

**Rationale:**

The majority of the scientific evidence on discogenic or spondylotic foraminal stenosis is lacking comparative data. Within this area, well-validated outcome instruments are mandatory and there is a need for comparative data to develop evidence based treatment recommendations. At this time, there are no evidence-based guidelines on the most appropriate surgical treatment strategy for cervical discogenic or spondylotic stenosis of the cervical neuroforamen. The results of this study will provide surgical treatment recommendations for patients with discogenic or spondylotic cervical foraminal stenosis and contribute to the understanding of its short- and long-term postoperative course.

**Objective**:

The study objectives are to compare clinical outcome (decrease of radiculopathy), complication rates and cost-effectiveness and work absenteeism of FOR to ACDF within the group of patients with a CRS due to soft disc compression or osteophyte compression of a cervical root.

**Study design:**

This study is a nationwide, prospective, multiCentre, researcher blinded, randomized controlled trial with a follow up period of in total 104 weeks.

**Study population:**

The trial will focus on patients with a monosegmental radicular pain syndrome due to a lateral or foraminal herniated disc or osteophyte, with cervical root compression. Patients with foraminal compression of the neuroforamen of C4,C5,C6 and C7 are included. Number of patients to include in the study: 308 patients.

**Intervention**

ACDF technique:

Microsurgical discectomy was performed through a ventral approach described by Smith and Robinson (1958). Procedure can be executed with microscope or loupe magnification. Exploration of the intervertebral disc and removal of bony spurs with a high-speed drill. Subsequently removal of the posterior part of the intervertebral space. The posterior ligament is dissected and removed with rongeurs. Subligamentous discal fragments are removed. The proximal part of the neuroforamen is inspected for discal remnants. If an osteophytic component is present, the uncovertebral joint is reduced to remove the osteophytic component. An intervertebral spacer is placed to keep height of the intervertebral disc space. No additional plate fixation is used.

FOR technique:

All patients are operated in prone position with the head fixated in a 3-point head holder. After determining the correct level on lateral radiograph, a vertical 4 cm midline incision is made, and the lateral lamina/medial facet joints are exposed. A retractor is placed adequately. Under the operating microscope or loupe magnification and after a second confirmation of the correct level, a partial hemilaminectomy and foraminotomy with partial facetectomy of the involved level is performed with high-speed drills. The percent of the facet resection is based on the extent of the foraminal pathology. In cases of pure soft discs, the proximal root is visualized adequately for removal of the compressing disc material. In cases of foraminal stenosis, bony decompression and skeletonization of the proximal root were performed carefully using a 4-mm diamond burr, small rongeurs, and dissectors.

**Main study parameters/endpoints:**

Primary outcome measure will be self-reported brachialgia by the Visual Analoge Scale/Odom’s criteria (4 point Likert scale for self-reported brachialgia). The secondary outcomes include Work Ability (Work Ability Index, single item), Quality of Life (EQ-5D), Neck Pain (VAS), Neck disability index (NDI), Complications and Productivity related costs (iPCQ) and medical costs (iMCQ).

**Nature and extent of the burden and risks associated with participation, benefit and group relatedness:** Patients are treated within the concept of “care as usual”. Both operative techniques are part of the neurosurgeons basic skills and training. Both operative techniques are indicated techniques to operate on cervical discal herniations. However, as differences in risks and clinical outcome between both procedures have never been studied in a comparative setting, their exact magnitude and nature is not yet known and therefore their assessment is one of the objectives of the study.

The burden for patients participating in this trial is low. Patients are asked to fill out questionnaires at baseline and after 6, 26, 52, 78 and 104 weeks. Time to fill out the questionnaires is approximately 30 minutes per follow up moment. There are no benefits compared to care as usual.

1. **INTRODUCTION AND RATIONALE**

Cervical radiculopathy is a disease with a significant impact on the patient’s quality of life. Most patients with a cervical radiculopathy belong to the labour force. Therefore temporary disability due to the radiculopathy often leads to health care problems and loss of productivity.

The incidence of the Cervical Radicular Syndrome (CRS) is estimated at 0,8 per 1000 inhabitants (1). This means for the Netherlands approximately 13000 new patients/year. In about 1700 cases an operation is executed (https://[www.kpmg.com/NL/nl/IssuesAndInsights/ArticlesPublications/Documents/PDF/Healthcare/Praktijk](http://www.kpmg.com/NL/nl/IssuesAndInsights/ArticlesPublications/Documents/PDF/Healthcare/Praktijk) variatie-rond-indicatiestelling-in-Nederlandse-ziekenhuizen.pdf). The revised Dutch CRS guideline (2010) states that a CRS existing longer than 2 months which does not respond to conservative treatment, can be indicated for surgical treatment (2). Various surgical approaches for the treatment of cervical disorders causing radiculopathy have been described. Scoville reported the successful removal of cervical discs with a laminectomy in 1944, furthermore, the posterior approach for the treatment of cervical radiculopathy has evolved into keyhole foraminotomy being popularized by Frykholm (3). Cloward, and Smith and Robinson first developed the anterior cervical discectomy approach in 1958 (9,10). Anterior discectomy has become the primary standard for treating cervical degenerative disc diseases resulting in stenosis of the central spinal canal or its neuroforamen. For lateral pathologies such as the foraminal soft disc protrusion or spondylitic stenoses of the neuroforamen, there is still controversy regarding the most adequate surgical approach. The literature in this matter consists of low-quality observational reports (see also systematic review). Both techniques are standard care, but the preference to use one technique above the other is partially based on surgeons’ s preference and not on clear evidence (5,8). The Anterior Cervical Discotomy with Fusion (ACDF) is the dominant option among surgeons in the Netherlands, but 4 arguments are available to challenge this dominance. 1. Morbidity data are in favor of posterior cervical foraminotomy (FOR). 2. Direct costs of FOR appear lower. 3. Indirect costs of FOR appear lower, because it may lead to faster work resumption, however this was analyzed in a suboptimal study design and results may not be generalizable to The Netherlands (9). 4. Clinical outcome for FOR appears non- inferior to ACDF, however this is not based on high quality research. This study proposes to analyze in a high quality design (multiCentre, investigator blinded, randomized controlled trial) the cost-effectiveness of the FOR technique compared to ACDF of 2 large groups of patients treated in a multiCentre setting for cervical monoradiculopathy caused by soft/osteophytic foraminal pathology.

HEALTH CARE EFFICIENCY PROBLEM

It is hypothesized that the FOR technique is efficient and lowers the direct and indirect (productivity) costs in comparison with the ACDF technique. The ACDF technique is executed with instrumentation (intervertebral cage), the FOR technique does not require instrumentation and therefore medical costs are lowered when using this technique. The expectation is that the FOR technique leads to earlier and better work participation after operation, however this is an assumption. It could be that because of more neck pain after the FOR treatment, patients remain longer at home and therefore work absenteeism is higher in the FOR treatment group. An important part of this study is to investigate work resumption among CRS patients operated by one of the surgical techniques.

THE INTERVENTION TO BE INVESTIGATED

The ACDF technique is performed through the anterior cervical plane by which the pre-vertebral cervical area can be reached. A discectomy is executed and nerve root decompression will be established. An intervertebral cage is routinely placed to reach fusion on the long term.

The FOR operation is an unroofing technique. By unroofing the foramen of the cervical root, the cervical root is decompressed. The FOR operation is a simple technique with low morbidity and no additional instrumentation (cage, plate, screws) is needed.

EXISTING EVIDENCE OF EFFECTIVENESS

High Risk of bias by morphology in which one of the two techniques is preferred (soft discus compression or osteophytic compression) and surgeon's bias concerning preference for ACDF is present within the literature, concerning the comparison of FOR against ACDF in patients with cervical radiculopathy. No randomized clinical trial (RCT) has been performed comparing both techniques concerning relief of arm pain, cost-effectiveness and work absenteeism.

INNOVATIVE CHARACTER

The innovative value of this trial is threefold:

1) There is insufficient high quality knowledge available which technique has the best outcome for a specific subgroup of patients with a CRS due to a lateral or foraminal compression. No randomized controlled trial has been executed within this group of patients.

2). Serious complications including morbidity by the FOR technique seem to be lower than for ACDF, however this is yet not studied in a RCT. If non-inferiority is present (decrease of radiculopathy) and morbidity is lower for the FOR technique, than the balance of choosing wisely should turn over to the FOR technique for decompressing the lateral/foraminal cervical nerve.

1. Hypothesized is, that the direct and indirect costs of surgical treatment of the CRS will be substantially

decreased due to FOR surgery. Cost-effectiveness of FOR compared to ACDF will be analyzed. This has never been done in an optimal controlled research situation. The proposed RCT will lead to objective cost effectiveness results and conclusions.

RELEVANCE FOR PRACTICE

If the FOR technique receives more scientific interest within the surgical community then patients with a lateral/foraminal compression of the cervical nerve root will be operated by this technique more frequently. Currently, in the Netherlands there is a preference for the ACDF due to its broader indication area. If FOR is cost-effective then the implication should be a modification of the Dutch Clinical Guidelines for the cervical radicular syndrome. Menzis, a Health Insurance Company, supports this project.

ANTICIPATED COST-EFFECTIVENESS

Replacing ACDF by FOR is expected to lower the costs of the treatment procedure by (€7.165 - €4.204) Dutch index price =DOT))= €2.961 per patient. Given that per year approximately 1300 (1:4 of 1700) patients have surgery for lateral root compression, this will lead to savings of €3.849.300 annually for Dutch society as a whole. Literature suggests that FOR may be as effective in relieving arm pain, the proposed study might confirm this. Whether or not this would translate into a better Quality of Life remains to be seen. A potentially very relevant component of a cost-effectiveness analysis from a societal perspective is costs related to reduced work participation. A previous study in patients with non-specific musculoskeletal disorders showed that indirect costs related to reduced work participation may take up to 80% of the total costs. Our literature review, however, revealed that these figures are unavailable for procedures under study in this proposal. The published research that has provided data on work productivity was performed within the context of the US armed services (4). The regulations were very specific (patients who were implanted a cage were mandatorily on sick leave for 6 weeks) and non- transferable to the Dutch social situation. Additionally, relevant data on work resumption are not available in hospital records in the Netherlands. Based on this, we have concluded that there are no data available on which to build a firm efficiency calculation (which would be another argument to perform the proposed study).

The following calculations, therefore, are based on clinical outcomes only, which is less speculative than work participation outcomes based on insufficiently generalizable literature or absent hospital records.

The presented studies of clinical effectiveness demonstrate a similar effect on relief of arm pain, but a lower chance of serious complications needing longer hospital stay or readmission. We assume a 10% difference between the ACDF and FOR, which means that serious complications would occur in 10% more of the cases in the ACDF, and 2 weeks extra hospital care attributed to a serious complication (10 work days). We also assume that of the 1300 patients treated per year, 50% will be among the working population, in a 75% part-time job on average. The standard mean costs of a working day in the Netherlands is €230 (11). Thus: 650 working patients x 10% with serious complications x 7.5 workdays lost per patient = 487.5 work days saved with the FOR procedure. This amounts to a total of €112.125.

1. **OBJECTIVES**

Primary Objective:

Is the posterior cervical foraminotomy (FOR) technique effective in comparison to the anterior cervical discectomy with fusion (ACDF) with regards to reduction of cervical radicular pain measured by the ODOM criteria (4 point Likert scale) and VAS arm pain.

Secondary Objective(s):

- 1. Does the FOR operation lead to faster return to work, after operation, in comparison with the ACDF technique,
  2. Does the FOR operation lead to a higher quality of life in comparison with the ACDF technique,
  3. Does the FOR operation lead to more or less neck disability and/or more or less postoperative neck pain in comparison with the ACDF technique,
  4. Does the FOR operation lead to less or more complications in comparison with the ACDF technique,
  5. Does the FOR operation lead to lower productivity losses and medical costs in comparison with the ACDF technique.
  6. What would be the budget impact of implementing the FOR technique, if it were to replace the ACDF technique as the recommended strategy for patients with a CRS due to soft disc compression or osteophyte compression of a cervical root

1. **STUDY DESIGN**

This study is a nationwide prospective, multiCentre, investigator blinded, randomized controlled trial with long term follow up of 24 months. Blinding of the data, the researcher is mandatory to avoid data interpretation bias. In this trial blinding of the patient or the surgeon is not feasible. Both the FOR (experimental group) and the ACDF(active control) have been established surgical techniques. From the literature, mainly retrospective or prospective cohort studies, it has been shown that both techniques are comparable in terms of clinical effect and complications. However these studies are prone to inclusion (surgeon) bias. Therefore an RCT is mandatory to adequately compare both techniques on efficacy. A non-inferiority trial design is chosen to show whether FOR (experimental) has at least as much efficacy as the ACDF technique (active control) or is worse by an amount less than 10%. It is hypothesized that FOR reduces costs, has fewer complications, leads to less work absenteeism, and leads to higher quality of life. All these parameters are secondary outcome measurements.

This project is a cooperation between the department of neurosurgery UMCG (34 patients/year) and 5 other Dutch neurosurgical departments (Martini Ziekenhuis Groningen (24 patients/year; primary investigator is dr. J. Koopmans), Radboud Universitair Medisch Centrum Nijmegen (23 patients/year; primary investigator is prof. dr. R. Bartels), Medisch Spectrum Twente (27/ patients/year; primary investigator is dr. B. Hoess), Atrium/Orbis ziekenhuis (22 patients/year; primary investigator is dr. H. van Santbrink) and the Medisch Centrum Haaglanden (24 patients/year; primary investigator is dr. M. Arts). The participating Centres are chosen because of their high volume of spine instrumented surgery and their familiarity with the two surgical procedures. All participating Centres have signed a Letter of Intent. A conservative inclusion ratio of 30% has been chosen so that after one year 154 patients can be included. The total number of patients to include after 2 years will be 308 patients

Study period

The inclusion of patients starts at December first 2015. Total follow-up is 2 years. At baseline (i.e. at enrolment, before the surgical procedure) and then at 6, 26, 52, 78 and 104 weeks after the surgical procedure patients will fill out questionnaires (see Table 1: Visit plan FACET study). After 78 weeks data the first data analysis will be carried out to proceed in fulfilling the ZonMW end of trial evaluation. ZonMW demands end of trial evaluation within 48 months after start of trial. However, the timeline of inclusion is 24 months and 24 months after surgical procedure patients will be asked to fill out the last questionnaire for final data analysis. Therefore the results of these data (24 months) will be published in an international journal. During these 24 months patients will be followed at different moments in time. For the first 4 visits patients will attend the hospital. For visits 5-8 no visits to the hospital are needed. Only web based questionnaires are to be filled out.

Description of the study visits;

If the patient is eligible for the study, the neurosurgeon will inform the patient orally and in writing. After one week the patient will inform the neurosurgeon whether or not he/she will participate in the study and the inform consent procedure will be fulfilled. Patients who fulfil the inclusion and exclusion criteria and signed the informed consent will be randomized to one of the two surgical procedures. Patients will proceed to have the operation either FOR or ACDF and will be admitted to the hospital one day before or at the day of surgical procedure. The following information will be assessed:

- Visit 1: Baseline evaluation after informed consent is signed
  - Medical and preoperative history
  - Clinical evaluation (signs, symptoms, strength, reflexes)
  - Web based patient self-assessment (questionnaires: arm pain VAS, neck pain VAS, WAI, EQ-5D, NDI, iMCQ, iPCQ)
  - Function X-ray of neck and arm
  - MRI and/or CT (If a patient recently (<6 months before operation) performed a Function X-ray or a MRI and/or CT, the result of these tests could be used as baseline value)
  - Report and record (S)AE (start after randomisation)
- Visit 2: Surgical Procedure (FOR or ACDF)
  - Surgical evaluation report (date, type, antibiotic prophylaxes, level of procedure, instruments, complications and (S)AE’s).
- Visit 3: Day of discharge
  - Clinical evaluation (signs, symptoms, strength, reflexes,)
  - Odom’s criteria)
  - Report and record (S)AE
- Visit 3.1: 1 week after discharge; arm and neck pain VAS (web based)
- Visit 3.2: 2 weeks after discharge; arm and neck pain VAS (web based)
- Visit 3.3: 3 weeks after discharge: arm and neck pain VAS (web based)
- Visit 3.4: 4 weeks after discharge: arm and neck pain VAS (web based)
- Visit 3.5: 5 weeks after discharge: arm and neck pain VAS (web based)
- Visit 4: 6 weeks after operation (± 1 week) (at outpatient clinic)
  - Clinical evaluation (signs, symptoms, strength, reflexes)
  - Odom’s criteria
  - Web based patient self-assessment on pain, quality of life and costs (questionnaires: arm pain VAS, neck pain VAS, WAI, EQ-5D, NDI, iPCQ, iMCQ)
  - Report and record (S)AE
  - Costs
- Visit 5: 26 weeks after operation (± 2 weeks) (patients do not attend the hospital)
  - Web based patient self-assessment on pain, quality of life and costs (questionnaires: arm pain VAS, neck pain VAS, WAI, EQ-5D, NDI, iPCQ, iMCQ)
  - Odom’s criteria
  - Report and record (S)AE
  - Costs
- Visit 6: 52 weeks after operation (± 4 weeks) (patients do not attend the hospital)
  - Web based patient self-assessment on pain, quality of life and costs (questionnaires: arm pain VAS, neck pain VAS, WAI, EQ-5D, NDI, iPCQ, iMCQ)
  - Odom’s criteria
  - Report and record (S)AE
  - Costs
- Visit 7: 78 weeks after operation (± 4 weeks) (patients do not attend the hospital)
  - Web based patient self-assessment on pain, quality of life and costs (questionnaires: arm pain VAS, neck pain VAS, WAI, EQ-5D, NDI, iPCQ, iMCQ)
  - Odom’s criteria
  - Report and record (S)AE
  - Costs
- Visit 8: 104 weeks after operation (± 4 weeks) (patients do not attend the hospital)
  - Web based patient self-assessment on pain, quality of life and costs (questionnaires: arm pain VAS, neck pain VAS, WAI, EQ-5D, NDI, iPCQ, iMCQ)
  - Odom’s criteria
  - Report and record (S)AE
  - Costs

1. **STUDY POPULATION**
   1. **Population (base)**

The trial will focus on patients with a monosegmental radicular pain syndrome due to a lateral or foraminal herniated disc or osteophyte, with cervical root compression. Patients with foraminal compression of the neuroforamen of C4,C5,C6 and C7 are included through the outpatient clinics of the participating hospitals. Neuroforamen compression of C8 is excluded because this patient population is more frequently operated from anteriorly. To see if the inclusion rate of 308 patients is feasible, in one of the outpatient clinics 25 patients with a cervical radiculopathy due to a cervical foraminal compression were asked whether they would participate in a surgical randomized controlled trial. More than 80% of the patients was willing to participate. Furthermore it was analyzed that with a low inclusion rate of 30% each year, we will be able to include 308 patients in 2 years.

- 1. **Inclusion criteria**

In order to be eligible to participate in this study, a subject must meet all of the following criteria:

- Age between 18 and 80 years.
- Cervical foraminal stenosis due to a soft disc component causing monoradiculopathy of C4, C5, C6, or C7 and requiring decompression of neuroforamen. (*Foraminal stenosis due to a soft disc component is defined as: 2/3 of the total discal component is located intraforaminally and a maximum of 1/3 of the total discogenic component is located medially, within the spinal canal. Radiculopathy is defined as pain, paralysis or paresthesia in corresponding nerve root distribution areas of C4, C5, C6,or C7,and must include at least arm or shoulder pain with minimum of 30 mm on a 100 mm visual analog scale)*.
- No response to conservative treatment for eight weeks or presence of progressive symptoms or signs of nerve root compression in the face of conservative treatment.
- Soft disc/Spondylitic foraminal stenosis (determined by MRI and CT and/or right or left oblique X- ray of the cervical spine) at the treatment level correlating to primary symptoms.
- Psychosocially, mentally, and physically able to fully comply with this protocol, including adhering to scheduled visits, treatment plan, completing forms, and other study procedures.
- Patient has sufficient mastery of the Dutch language to fill out the questionnaires.
- Signed and dated informed consent document prior to any study-related procedures
  1. **Exclusion criteria**

A potential subject who meets any of the following criteria will be excluded from participation in this study:

- Multisegmental CRS.
- Median located disc protrusion or osteophytic protrusion.
- Foraminal compression of C8.
- Spinal cord compression with clinical myelopathy.
- Radiological myelopathy.
- History of cervical spine surgery.
- Malignant obesity (BMI > 30).
- Osteoporosis / chronic use of corticosteroids.
- ASA 4 and 5 patients (serious ill patients).
- Pregnancy
- Active malignancy
- Abundant use of alcohol, drugs, narcotics and recreational drugs.
- Contra-indications for anesthesia or surgery
- Patient has used another investigational drug or device within the 30 days prior to surgery
- Incapability to speak and write the Dutch language
  1. **Sample size calculation**

The sample size analysis is based on the manuscript of Dohrman et al (12). Based on this review on cohort studies an overall success rate of 87% for both groups was detected. Based on the current guidelines there is evidence that FOR is not worse in comparison with ACDF concerning decrease in arm pain. Therefore the assumption is that there will not be a statistically significant difference between the two surgical techniques concerning the primary outcome measure (relief of arm pain). Therefore with a binary non inferiority calculation with a success rate of 87%, alpha 0.05. power of 0.8 and a non-inferiority margin (delta) of 10%, and including a 10% drop outs, we need 154 per arm, totally 308 patients.

1. **TREATMENT OF SUBJECTS**

The interventions in this trial are standard surgical procedures which are performed for many years in all neurosurgical departments in hospitals in the Netherlands and beyond.

**5.1 Investigational treatment**

ACDF technique:

Microsurgical discectomy is performed through a ventral approach described by Smith and Robinson (1958). Procedure can be executed with microscope or loupe magnification. Exploration of the intervertebral disc and removal of bony spurs with a high-speed drill. Removal of the posterior part of the intervertebral space. The posterior ligament is dissected and removed with rongeurs. Subligamentous discal fragments are removed. The proximal part of the neuroforamen is inspected for discal remnants. If an osteophytic component is present, the uncovertebral joint is reduced to remove the osteophytic component. An intervertebral spacer is placed to keep height of the intervertebral disc space. No additional plate fixation is used.

FOR technique:

All patients are operated in prone position with the head fixated in a 3-point head holder. After determining the correct level on lateral radiograph, a vertical 4 cm midline incision is made, and the lateral lamina/medial facet joints are exposed. A retractor is placed adequately. Under the operating microscope or loupe magnification and after a second confirmation of the correct level, a partial hemi-laminectomy and foraminotomy with partial factectomy of the involved level is performed with high-speed drills. The percentage of the facet resection is based on the extent of the foraminal pathology. In cases of pure soft discs, the proximal root is visualized adequately for removal of the compressing disc material. In cases of foraminal stenosis, bony decompression and skeletonization of the proximal root are performed carefully using a 4-mm diamond burr, small rongeurs, and dissectors.

1. **METHODS**
   1. **Study parameters/endpoints**
      1. **Main study parameter/endpoint**

The main study parameter is to compare the clinical outcome (decrease in radiculopathy assessed by (Visual Analogue Scale for self-reported brachialgia /Odom’s criteria) between patients operated with the FOR technique or with the ACDF technique during 24 months of follow up.

Secondary study parameters are:

1. Changes in work ability (Work Ability Index, single item) during 24 months of follow up between the two groups.
2. Changes in quality of life (EQ-5D) during 24 months of follow up between the two groups.
3. Changes in neck pain (VAS during 24 months of follow up between the two groups .
4. Changes in Neck Disability Index (NDI) during 24 months of follow up between the two groups .
5. Number or percentage of complications in the short (30 days) and long term period (104 weeks) between the two groups
6. Cost-effectiveness (104 weeks)
7. Budget impact (extrapolated to 5 years)
   1. **Randomisation, blinding and treatment allocation**

Enrolment will be performed in the 6 Centres in the Netherlands. Patients are randomized to either FOR or ACDF. Randomization will be executed per patient per Centre by web based block randomization after the informed consent procedure is fulfilled. The Trial Coordination Centre will facilitate the randomization. Number of inclusions per Centre is described in the patient enrolment plan as described in section 6.3 (study procedures).

- 1. **Study procedures**

If the patient seems to be eligible for a surgical procedure, the patient will be invited for a consultation with the neurosurgeon. He/she will inform the patient about the surgical procedure and the possibility to participate in the FACET study. The neurosurgeon will provide the patient with the study-information.

After one week, the patient can let the neurosurgeon know if he/she wants to participate in the study. Subjects who fulfil the inclusion and exclusion criteria and signed the informed consent will proceed to have the surgical procedure. Before the procedure patients will be clinically evaluated by the surgeon and anesthesiologist to be sure the surgical procedure can be performed. Which procedure is decided by randomization. Randomization will take place before the surgical procedure will be performed and after informed consent is signed. The surgeon and the patient will know to which procedure the patient is randomized. The patient will be admitted to the hospital before the procedure and will be monitored by standard hospital practice before and after the procedure. The patient will be discharged from the hospital if he/she is stable according to the responsible physician. Normally a hospital admission for this procedure takes 2-3 days. The patient will be reviewed at the outpatient clinic at 6 weeks after the surgical procedure, which is standard care. At baseline, 6, 26, 52, 78 and 104 weeks after the surgical procedure the patient is invited to fill out a web based self-administered questionnaire to monitor work ability, arm and neck pain, quality of life, complications and costs according to the visit plan (table 1).

**NL54380.042.15 FACET study**

Table 1: Visit plan FACET study

|  | Visit plan |  |  |  |  |  |  | | | |
| --- | --- | --- | --- | --- | --- | --- | --- | --- | --- | --- |
|  |  | **Visit 1** Baseline (preoperative) | **Visit 2**  Procedure | **Visit 3**  Discharge | **Visit 3.1**  **t/m 3.5**  week 1-5 after OK | **Visit 4**  6 weeks after OK | **Visit 5**  26 weeks after OK | **Visit 6**  52 weeks after OK | **Visit 7**  78 weeks after OK | **Visit 8**  104 weeks after OK |
| 1 | Informed consent | x |  |  |  |  |  |  |  |  |
| 2 | Function X-ray | x |  |  |  |  |  |  |  |  |
| 3 | MRI and/or CT | x |  |  |  |  |  |  |  |  |
| 4 | Preoperative history | x |  |  |  |  |  |  |  |  |
| 5 | Clinical evaluation | x |  | x |  | x |  |  |  |  |
| 6 | Randomization | x |  |  |  |  |  |  |  |  |
| 7 | Operative detail |  | x |  |  |  |  |  |  |  |
| 8 | Patient self- assessment* | x |  |  | X (VAS arm and neck pain) | x | x | x | x | x |
| 9 | (S)AE recording and reporting |  | x | x |  | x | x | x | x | x |
| 10 | Costs | x |  | x |  | x | x | x | x | x |

*Patient self-assessment questionnaires will be web based and assessed at baseline, 6, 26, 52,78 and 104 weeks after surgical procedure

Evaluations during the study

- 1. Signing informed consent.

Prior to the start of the study a signed and dated informed consent will be obtained from the patient.

- 1. Function X ray

An X-ray of the neck and arm will be performed and is standard care.

- 1. MRI and/or CT scan

An MRI and/or CT scan will be performed to assess inclusion and exclusion criteria. These scans are part of normally care for these patients. If one of these tests is made 6 months or less before the surgical procedure, the information can be used an no new scan has to be made.

- 1. Preoperative history

From standard care procedures preoperative history is obtained including length, weight, ASA score, number of months/years of neck and arm pain, diagnosis, number of months the patient is diagnosed for cervical soft/osteophytic disc disease, signs and symptoms, other significant illnesses, pain medication, smoking history, use of NSAIDs. Medical charts will be used to verify this information.

- 1. Clinical evaluation

At baseline, discharge and 6 weeks follow up, information on signs and symptoms, reflexes and strength, will be assessed which is according to the standard care.

- 1. Randomization

If a patient is eligible and after informed consent completion the patient will be randomized to undergo one of the two procedures (FOR or ACDF).

- 1. Operative detail

During the operation information about the operative procedure will be obtained from the medical record of the patient such as date and type of procedure, which level is operated, use of implants, and the appearance of complications.

- 1. Patient self-assessment

The self-reported brachialgia (VAS), ODOMs criteria, Work Ability Index (WAI), quality of life (EQ-5D), neck pain (VAS), Neck Disability Index (NDI), Productivity Costs (iPCQ) and Medical Consumption (iMCQ) will be assessed by using web based questionnaires. Filling out these questionnaires takes about 30 minutes per follow up time and is additional information due to the study.

- 1. (S)AE reporting

Throughout the study (S)AE’s have to be recorded and reported according to the protocol.

- 1. Costs

Cost data will be collected in an electronic CRF and by questionnaires. Included costs will be those of the surgical procedure (either FOR or ACDF), hospital care (including costs for treating complications), medication related to the diagnosis, outpatient visits, GP visits, home care, and productivity costs due to absence from work. (Productivity Costs Questionnaire iPCQ, Medical Consumption Questionnaire iMCQ) . Unit prices will be determined according to Dutch guidelines (6).

In total 308 patients will be included in 6 centres in the Netherlands. In table 2 the enrolment schedule of the participating centres is showed.

Table 2: Patient enrolment schedule participating Centres.

| Patient enrolment schedule participating Centres | | | | | | | | | | | | | |
| --- | --- | --- | --- | --- | --- | --- | --- | --- | --- | --- | --- | --- | --- |
|  | Dec 2015 | Jan 2016 | Feb 2016 | March 2016 | April 2016 | May 2016 | June 2016 | July 2016 | Aug 2016 | Sept 2016 | Oct 2016 | Nov 2016 | Total  enrolment 1 yr |
|  |  |  |  |  |  |  |  |  |  |  |  |  |  |
| UMCG | 3 | 3 | 3 | 3 | 3 | 3 | 3 | 3 | 2 | 2 | 3 | 3 | 34 |
| Martini  ziekenhuis | 2 | 2 | 2 | 2 | 2 | 2 | 2 | 2 | 2 | 2 | 2 | 2 | 24 |
| Radboud UMC | 2 | 2 | 2 | 2 | 2 | 2 | 2 | 2 | 2 | 1 | 2 | 2 | 23 |
| Atrium/Orbis ziekenhuis | 2 | 2 | 2 | 2 | 2 | 2 | 2 | 2 | 1 | 1 | 2 | 2 | 22 |
| MS Twente | 2 | 2 | 2 | 2 | 2 | 2 | 2 | 2 | 2 | 3 | 3 | 3 | 27 |
| MC  Haaglanden | 2 | 2 | 2 | 2 | 2 | 2 | 2 | 2 | 2 | 2 | 2 | 2 | 24 |
|  | | | | | | | | | | | | | 154 |
|  | Dec 2016 | Jan 2017 | Feb 2017 | March 2017 | April 2017 | May 2017 | June 2017 | July 2017 | Aug 2017 | Sept 2017 | Oct 2017 | Nov 2017 | Total  enrolment 2 yr |
| UMCG | 3 | 3 | 3 | 3 | 3 | 3 | 3 | 3 | 2 | 2 | 3 | 3 | 34 |
| Martini ziekenhuis | 2 | 2 | 2 | 2 | 2 | 2 | 2 | 2 | 2 | 2 | 2 | 2 | 24 |
| Radboud UMC | 2 | 2 | 2 | 2 | 2 | 2 | 2 | 2 | 2 | 1 | 2 | 2 | 23 |
| Atrium/Orbis  ziekenhuis | 2 | 2 | 2 | 2 | 2 | 2 | 2 | 2 | 1 | 1 | 2 | 2 | 22 |
| MS Twente | 2 | 2 | 2 | 2 | 2 | 2 | 2 | 2 | 2 | 3 | 3 | 3 | 27 |
| MC  Haaglanden | 2 | 2 | 2 | 2 | 2 | 2 | 2 | 2 | 2 | 2 | 2 | 2 | 24 |
|  | | | | | | | | | | | | | 154 |
|  | | | | | | | | | | | | Total | 308 |

- 1. **Withdrawal of individual subjects**

Subjects can leave the study at any time for any reason if they wish to do so without any consequences. The investigator can decide to withdraw a subject from the study for urgent medical reasons.

- - 1. **Replacement of individual subjects after withdrawal**

The goal is to reach 308 patients. When a patient withdraws from the study, he/she will not be replaced since 10% loss of inclusion is calculated within the power calculations of the trial.

- - 1. **Follow-up of subjects withdrawn from treatment**

There will be no follow up of patients who are withdrawn from the study before the surgical procedure has taken place. If patients are withdrawn after they have received the treatment there will be follow up of medical data when necessary without bothering and with permission of the patient. Data of patients after withdrawal will be used and analysed with the intention to treat principle.

- - 1. **Premature termination of the study**

Premature suspension of the study will only occur in specific situations:

- Inclusion ratio: if inclusion one year after the last Centre has included its first patient is in total more than 50% below the amount of patients intended to include in all 6 centres, the study will be terminated.
- Patient health: there are no adverse reactions known on which termination of this study will be mandatory. Both surgical procedures are standard care.

The Medical Ethics committee will be informed about the suspension.

1. **SAFETY REPORTING**
   1. **Section 10 WMO event**

In accordance to section 10, subsection 1, of the WMO, the investigator will inform the subjects and the reviewing accredited METC if anything occurs, on the basis of which it appears that the disadvantages of participation may be significantly greater than was foreseen in the research proposal. The study will be suspended pending further review by the accredited METC, except insofar as suspension would jeopardise the subjects’ health. The investigator will take care that all subjects are kept informed.

- 1. **AEs, SAEs and SUSARs**
     1. **Adverse events (AEs)**

Adverse events are defined as any undesirable experience occurring to a subject during the study, whether or not considered related to intervention. The following complications are known by the researchers and are described in the literature. These complications will be listed as adverse events. No other adverse events will be administrated. The complications that will be assessed are the following:

- Death
- Thrombosis
- Pulmonary embolism
- Urinary retention
- Post-operative bleeding / Hematoma
- Post-operative wound infection
- Nerve root injury
- Dural tear
- Post-operative cerebrospinal fluid leakage
- Implant malposition
- Vascular injury
- Blood loss
- Urinary infection
- Spinal cord injury
- Esophagus injury
- Hoarseness
- Pneumonia
- other

Complications will be written down in the eCRF as adverse events.

- - 1. **Serious adverse events (SAEs)**

A serious adverse event is any untoward medical occurrence or effect that at any dose:

- results in death;
- is life threatening (at the time of the event);
- requires hospitalisation or prolongation of existing inpatients’ hospitalisation;
- results in persistent or significant disability or incapacity;
- is a congenital anomaly or birth defect;
- Any other important medical event that may not result in death, be life threatening, or require hospitalization, may be considered a serious adverse experience when, based upon appropriate medical judgement, the event may jeopardize the subject or may require an intervention to prevent one of the outcomes listed above.

Reporting procedure applies to all SAE's occurring from the time a subject gives consent until the end of the study. A life threatening SAE, or SAE with death as a result, must be reported within 7 days after the local investigator has been informed. Other SAEs must be reported within 15 days. The study coordinator is responsible for reporting SAEs at CCMO module ‘ToetsingOnline’.

For individual sites, the primary investigator completes the SAE report providing as much

detailed information as known and relevant to the event. The primary investigator sends the complete SAE form by e-mail to the UMCG study coordinator within 24 hours of discovery of the event.

Thus, the coordinating investigator will be notified by email or telephone within 24 hours after discovery of the event. Using the CCMO module ‘ToetsingOnline’, all SAEs will be reported to the

CCMO and central METC. The reporting will occur within 15 days after the investigator has first received information on the SAE. For fatal or life-threatening cases a preliminary report will be offered within 7 days followed by a complete report within 8 days.

The following SAE’s do not require immediate reporting but will be reported once yearly in line listings to the accredited METC that approved the protocol:

- Elective hospitalization for pre-existing conditions that have not been exacerbated by trial treatment
- A hospitalization which was planned before the subject consented for study participation and where admission did not take longer than anticipated
  - 1. **Follow-up of adverse events**

All AEs will be followed until they have abated, or until a stable situation has been reached. Depending on the event, follow up may require additional tests or medical procedures as indicated, and/or referral to the general physician or a medical specialist.

- 1. **Safety Committee**

Because of the low to moderate risk for participants in the study a Data Safety Monitoring Board is not needed. To ascertain that the study is safe for participants, the study coordinator will report monthly a list of all (S)AE’s to the Principle Investigator (PI). In case the PI ascertain that more (S)AE’s occur in one of the two arms compared to the other arm, he will ask three independent physicians to form a safety committee and judge the safety of the subjects of the study. Detailed information on the trial and the (S)AE’s will be provided to the safety committee.

1. **STATISTICAL ANALYSIS**
   1. **Primary study parameter(s)**

Due to the requirements of ZONMW the analyses will be done at 78 weeks, however end of trial analyses will be done at 104 weeks and these data will be published. The primary endpoint (Odom's criteria and self-reported brachialgia) will be analysed as appropriate depending on data distribution with a one sided

0.05 level of significance (non-inferiority). Detailed descriptive statistics will be provided for the data collected and 95% confidence intervals will be calculated for all relevant estimates. Clinical follow-up data will be analysed by analysis of covariance (ANCOVA) or generalized model alternatives for categorical or semi quantitative data. Changes within the treatment groups over time as well as differences between groups will be assessed by intention-to-treat analyses. Also the primary analysis will follow the per protocol principle. Sensitivity analysis will be provided to evaluate robustness of the results with regard to unexpected circumstances (for example, impact of ‘cross-over’ patients who are not treated as randomized but are required to be analysed as randomized and centre effects).

- 1. **Secondary study parameter(s)**

Secondary endpoints will be analysed in an exploratory manner at a two-sided significance level of 5%. Safety and tolerability parameters will be analysed descriptively.

Analysis of time-dependent probabilities of critical events will be performed using the Kaplan-Meier method. Furthermore, multivariate event analyses will be performed using Cox proportional hazard regression models. In addition, for the purpose of a supportive sensitivity analysis, multiple imputation procedures will be applied.

COST-EFFECTIVENESS ANALYSIS

The cost-effectiveness analysis will be performed alongside the proposed clinical trial to assess the cost- effectiveness of FOR versus ACDF. There will be two separate outcome measures for the cost- effectiveness analysis, resulting in two incremental cost-effectiveness ratios (ICERs) for FOR as compared to ACDF, the first is incremental costs per extra percentage of patients with arm pain relief, the second is incremental costs per Quality Adjusted Life Year (QALY) gained. The analysis will be performed taking a societal perspective. The time horizon will be equivalent to the full follow-up of the clinical study, which is 24 months. According to pharmaco-economic guidelines (6) discounting will be applied for costs (4%) and effects (1.5%) in the second year. A number of sensitivity analyses will be performed to identify the impact of variables such as the costs of the FOR and ACDF procedures, resource use, and effect size, on cost-effectiveness. A cost-effectiveness acceptability curve will be constructed, based on bootstrap simulations, showing the probability of FOR being cost-effective compared to ACDF at varying levels of the willingness to pay, for either one additional percentage of patients with arm pain relief, or one additional QALY.

PATIENT OUTCOME ANALYSIS

The first outcome for the cost-effectiveness analysis is the percentage of patients with arm pain relief, which will be measured and analysed by the VAS, Odom's criteria, neurological testing and the NDI for the extent of neck pain. The second outcome is QALYs, which are assessed by means of the EQ-5D questionnaire (11) EQ-5D scores will be converted into health state utilities, ranging between 0 and 1, with a higher utility indicating a better health-related Quality of Life (QoL). These utilities will be multiplied with follow-up time spent in that particular health state (area under the curve) to eventually convert into QALYs.

BUDGET IMPACT ANALYSIS (BIA)

Based on the results of the clinical study and the cost-effectiveness analysis, a budget impact analysis will be performed to inform decision makers on the financial consequences of implementing FOR as treatment of first choice for cervical soft disc/osteophytic disease in the Dutch health care system. The BIA will be performed according to the principles of the ISPOR task force (7) and from both the perspective of the government (societal and BKZ – budgettair kader zorg) as well as a third party payer/healthcare insurers perspective. The trial results will be extrapolated, by means of a simple model, from a time horizon of 24 months to 5 years, and for the entire Dutch population concerned. The extrapolation will assume a constant incidence of cervical soft disc /osteophytic disease. Also, we expect that the proportion of cases eligible for surgical intervention will be stable over time. Therefore the extrapolation will be linear. Sensitivity analyses will be performed on relevant parameters such as the eventual substitution rate of FOR versus ACDF (may not be 100%), the uptake of FOR with time, costs of the procedures and other cost items. We will assume that current usual care already consists of a mix of ACDF and FOR, i.e. 90% and 10%, respectively.

1. **ETHICAL CONSIDERATIONS**
   1. **Regulation statement**

It is the responsibility of the investigator that this study will be conducted in accordance with the Declaration of Helsinki and in compliance with the Medical Research Involving Human Subjects Act (WMO).

- 1. **Recruitment and consent**

Patients will be informed in person (by the neurosurgeon and they will receive the written information concerning this trial. They will be informed about the procedure in this study, the duration and possible risks. Patients are allowed to take one week to make the decision whether they will take part in this study. The patient will be required to sign the written informed consent form if he wants to take part in this study. The right to the participant to refuse to participate without giving reasons will be respected. All participants are free to withdraw at any time from the protocol without giving reasons and without prejudicing further treatment.

- 1. **Benefits and risks assessment, group relatedness**

As described in chapter 5, the procedure is part of standard care for many years in most hospitals. It was shown that the risk of complications due to the procedure in both techniques are equal and due to the risk of the operation itself and is not due to the study procedures. The surgical procedure itself does not appreciably increase the risk of serious adverse events beyond the risk of undergoing the surgical procedure. Therefore we can conclude that the FACET study is a low to moderate risk study (according to the NFU risk classification) in which the benefits are high. In total 80-90 % of the patients are relieved of their cervico brachialgia after surgery.

- 1. **Compensation for injury**

The sponsor/investigator has a liability insurance which is in accordance with article 7 of the WMO. The sponsor (also) has an insurance which is in accordance with the legal requirements in the Netherlands (Article 7 WMO). This insurance provides cover for damage to research subjects through injury or death caused by the study.

The insurance applies to the damage that becomes apparent during the study or within 4 years after the end of the study.

1. **ADMINISTRATIVE ASPECTS, MONITORING AND PUBLICATION**
   1. **Handling and storage of data and documents**

The investigator will permit access to all patient source documents including original medical records by applicable regulatory authorities or the Medical Ethics Committee (METc), and consent to this documentation being available for review on request. The CRF will be completed for every patient and will capture all relevant patient information including details of any complications or adverse events. The CRF will be completed by properly trained and authorized personnel and each CRF will be approved by the investigator ensuring the data accurately reflects the patients’ clinical condition. The clinical data in the CRF will be entered into a computerized database for secure storage and in preparation for analysis.

Data will be handled confidentially. Where it is necessary to be able to trace data to an individual subject, a subject identification list is used to link the data to the subject. This code will not be based on the patient initials and birth-date, but will be based on the study site followed by the number of entering in the study (for example, the first patient included in the UMCG get number 10-001). The key to the code will be safeguarded by the investigator at the participating sites. The handling of personal data will comply with the Dutch Personal Data Protection Act (Wbp).

- 1. **Monitoring and Quality Assurance**

Monitoring activities will be performed on the basis of the Monitoring Plan. We will arrange 1 investigator meeting before actual start of the study and 2 or 3 monitor visits per site depending on the inclusion rate and findings of the monitor ( in total 15 monitor visits) will be performed. Monitoring will be performed in compliance with Good Clinical Practice and applicable national regulations.

No initiation visits (per site) will be performed. Instead, one investigator meeting in the Centre of The Netherlands will be organized. Monitoring objectives, tasks and issues will be presented by the study coordination institute (TCC).

**Regular monitoring visits:**

Monitoring of the main CRF data will be done on the basis of source data verification of a sample of CRFs (an estimated sample size is 10%). Main focus of the monitoring to be executed is related to the enrollment criteria, the informed consent procedure, safety parameters and the primary endpoint. Exact details will be documented in the monitor plan.

Further activities will involve checks of the Site File, study specific procedures and contact with the site before each visit and contact after the visit regarding unresolved queries.

The sponsor will receive comprehensive monitor reports for every visit within 14 days.

**Close-out visits:**

There will be no separate close-out visits. During the last monitoring visit, the site will be instructed on how to prepare their documentation in such a way that it is ready for long-term archiving. They will also be told about their obligations to keep the applicable documents properly archived over a period of 15 years.

- 1. **Amendments**

Amendments are changes made to the research after a favourable opinion by the accredited METC has been given. All amendments will be notified to the METC that gave a favourable opinion.

Non-substantial amendments (such as typing errors, and administrative changes like changes in names, telephone numbers and other contact details of involved persons mentioned in the submitted study documentation) will not be notified to the accredited METC and the competent authority, but will be recorded and filed by the sponsor.

- 1. **Annual progress report**

The sponsor/investigator will submit a summary of the progress of the trial to the accredited METC once a year. Information will be provided on the date of inclusion of the first subject, numbers of subjects included and numbers of subjects that have completed the trial, serious adverse events/serious adverse reactions, other problems, and amendments.

- 1. **End of study report**

The investigator will notify the accredited METC of the end of the study within a period of 8 weeks. The end of the study is defined as the last patient’s last visit.

In case the study is ended prematurely, the investigator will notify the accredited METC within 15 days, including the reasons for the premature termination. Within one year after the end of the study, the investigator/sponsor will submit a final study report with the results of the study, including any publications/abstracts of the study, to the accredited METC.

- 1. **Public disclosure and publication policy**

Since this is an investigator initiated trial, and no commercial sponsor is involved, no disclosures are warranted. Results will be published in peer reviewed journals and discussed at national and international meetings.

1. **STRUCTURED RISK ANALYSIS**

This is a low to moderate risk study according to the risk classification of the NFU. Both techniques are standard care. Both techniques are executed on large scale within the neurosurgical community since 1949 (FOR) and 1953 (ACDF).

- 1. **Potential issues of concern**

Participation in this clinical study may expose the patient to the following potential risks associated with the two surgical procedures. Due to the surgical procedure the following complications or adverse events could be apparent:

- Death
- Thrombosis
- Pulmonary embolism
- Urinary retention
- Post-operative bleeding / Hematoma
- Post-operative wound infection
- Nerve root injury
- Dural tear
- Post-operative cerebrospinal fluid leakage
- Implant malposition
- Vascular injury
- Blood loss
- Urinary infection
- Spinal cord injury
- Esophagus injury
- Hoarseness
- Pneumonia
- other

The chance of the occurrence of these complications in ACDF is 3-15% and in FOR this is 3-7%. The potential risks are considered rather small but the impact could be high depending on the kind of complication. No extra risk due to the study is anticipated and therefore this study is classified as low to moderate. Comparing these risks to the efficacy data to date, it appears that the benefit of the surgical procedures outweighs the risks.

Anesthesia

The potential risks associated with deep sedation are:

- Allergic reaction
- Nausea or vomiting
- Headache
- Pain and/or bruising at injection sites
- Sore or dry throat and lips
- Blurred and double vision
- Fever

However trained professionals with extensive experience who routinely administer general anaesthesia or local anaesthesia with deep sedation to patients will be responsible for the induction and associated monitoring required for this study.

Radiation

By participating in the study patients will be exposed to radiation during one function X-ray and a MRI and/or CT scan at baseline. The estimated exposure is minimal and will have no consequences for the participating patients.

- 1. **Synthesis**

This study is considered as a low to moderate risk study. It is shown that both surgical techniques are part of care as usual for many years in the participating hospitals and will be conducted by neurosurgeons with excellent expertise and experience with both techniques. The risks associated with the study are largely attributed to the surgical procedure itself and not to the study procedures. Therefore participating in the study does not increase the risk of serious adverse events beyond the risk of undergoing the surgical procedures. We expect that the benefits for the patients will be high and outweigh the risks*.*

1. **REFERENCES**
2. Radhakrishnan K et al.Epidemiology of cervical radiculopathy. A population-based study from Rochester, Minnesota, 1976 through 1990. Brain 1994.
3. Nederlands Vereniging voor Neurochirurgie. Richtlijn Behandeling van cervicaal radiculair syndroom ten gevolge van een cervicale Hernia Nuclei Pulposi. 2010.
4. Frykholm R. Deformitie od dural pouchesnnnnbrachial neuralgia. J Neurosurgery 1947,4:403-413.
5. Tumialan LM et al. Management of unilateral cervical radiculopathy in the military: the cost effectiveness of posterior cervical foraminotomy compared with anterior cervical discectomy and fusion. Neurosurg Focus 2010.
6. Gebremariam L et al. Evaluation of treatment effectiveness for the herniated cervical disc: a systematic review. Spine 2012.
7. L.Hakkaart- van Roijen et al. Handleiding voor kostenonderzoek. Methoden en standaard kostprijzen voor economische evaluaties in de gezondheidszorg. Geactualiseerd versie 2010. CvZ, Diemen
8. Sullivan SD, Mauskopf JA, Augustovski F, et al. Principles of good practice for budget impact analysis II: Report of the ISPOR Task Force on Good Research Practices – Budget Impact Analysis. Value Health 2014:17:5-14.
9. Fehlings et al. Posterior cervical foraminotomy for the treatment of cervical radiculopathy.Journal of Neurosurgery: Spine, April 2009.
10. Cloward RB. The anterior approach...cervical disks 1958.J Neurosurgery Spine 2007,6:496-511.
11. Smith GW, Robinson RA. The treatment of certain cervical-spine disorders by anterior removal of the intervertebral disc and interbody fusion. J Bone Joint Surg Am 1958,40-A:607-624.
12. EuroQol Group. EuroQol--a new facility for the measurement of health-related quality of life. Health Policy. 1990 Dec;16(3):199-208.
13. George J. Dohrmann Joseph C. Hsieh. Long-Term Results of Anterior versus Posterior Operations for Herniated Cervical Discs: Analysis of 6,000 Patients. Med Princ Pract 2014;23:70–73.

**Table 1. Time points and calculations for iMCQ and iPCQ**

| **Time points** | **Questionnaire** | **Recall period** | **Multiplication** |
| --- | --- | --- | --- |
| 6 weeks after surgery | iMCQ | 6 weeks | none |
|  | iPCQ | 6 weeks | none |
| 6 months after surgery | iMCQ | 3 months | x 1.5 |
|  | iPCQ | 4 weeks | x 5 |
| 12 months after surgery | iMCQ | 3 months | x 2 |
|  | iPCQ | 4 weeks | x 6.5 |
| 18 months after surgery | iMCQ | 3 months | x 2 |
|  | iPCQ | 4 weeks | x 6.5 |
| 24 months after surgery | iMCQ | 3 months | x 2 |
|  | iPCQ | 4 weeks | x 6.5 |

*Abbreviations:* the Institute for Medical Technology Assessment Medical Consumption Questionnaire (iMCQ), the Institute for Medical Technology Assessment Productivity Cost Questionnaire (iPCQ)

**Table 2. Baseline healthcare resource use and productivity losses.**

|  |  | **Costs in Euro** |  |
| --- | --- | --- | --- |
|  | **Posterior Surgery^a^** | **Anterior Surgery** | **Difference (95% CI)** |
| General practitioner | 85 | 82 | -3 (-30 to 23) |
| Physiotherapist | 141 | 199 | 58 (-18 to 135) |
| Company doctor | 31 | 28 | -3 (-20 to 13) |
| Other primary care^b^ | 35 | 38 | 3 (-35 to 41) |
| Care at home | 0 | 44 | 44 (-10 to 98) |
| Neurosurgeon | 122 | 142 | 20 (-9 to 49) |
| Other outpatient clinic visits | 167 | 134 | -33 (-85 to 19) |
| Pain medication | 33 | 31 | -2 (-18 to 15) |
| Emergency department^c^ | 102 | 25 | -77 (-129 to -25) |
| Hospital admissions | 112 | 90 | -23 (-136 to 91) |
| Additional health care costs^d^ | 80 | 192 | 112 (-4 to 228) |
| **Total health care costs** | **998** | **1,223** | **225 (-141 to 591)** |
| Sick leave before surgery, no, (%)  Sick leave > 85 days, no, (%)  Total days of sick leave | 45 (56)  22 (49)  97 | 55 (61)  20 (36)  110 | 13 (-36 to 63) |
| Absenteeism paid work  *Without friction cost method ^e^*  Absenteeism paid work  *With friction cost method* | 3,154  755 | 4,516  2,161 | 1,362 (-837 to 3,561)  1,405 (483 to 2,328) |
| Presenteeism paid work | 2,542 | 1,927 | -614 (-3,262 to 2,034) |
| Absenteeism unpaid work | 274 | 348 | 74 ( -145 to 294) |
| **Total societal costs^f^** | **3,571** | **4,493** | **922 (-1,944 to 3,788)** |

*Abbreviations: Confidence Interval (CI), Number of patients (no.)*

^a^ Percentages may not total 100, because of rounding. Data was missing for 7 (6%) patients in the posterior group and 7 (6%) in the anterior group. Values are presented as mean costs, expressed in rounded Euros.

^b^ Other primary care includes visits to social workers, occupational therapists, speech therapists, dieticians, alternative therapists and psychologists.

^c^ Emergency department includes visits to the emergency department and transportation to the hospital by ambulance.

^d^ Additional health care costs include daycare or admissions to a health institution other than a hospital, rehabilitation Centre or psychiatric institution.

^e^ The ‘friction cost method’ assumes that after a period of 85 days of complete absence, an employee gets replaced by another worker, and productivity would be restored.

**^f^** Total societal costs include absenteeism with the use of the ‘friction cost method”.

**Table 3. Cost differentiation with number of patients reporting costs**

|  | **Posterior Surgery**  **n=119** | **Anterior Surgery**  **n=124** | **Difference (95% CI)** |
| --- | --- | --- | --- |
| General practitioner | 634 | 613 | 28 |
| *n reported* | 71 | 78 |  |
| Physiotherapist  *n reported* | 1,179  50 | 1,450  55 | -355 |
| Company doctor | 158 | 182 | -31 |
| *n reported* | 47 | 41 |  |
| Other primary care^b^  *n reported* | 693  30 | 804  28 | -146 |
| Care at home^c^  *n reported* | 886  0 | 1,450  6 | -739 |
| Neurosurgeon  *n reported* | 338  85 | 298  78 | 52 |
| Other outpatient clinic visits  *n reported* | 734  49 | 1,062  47 | -430 |
| Pain medication  *n reported* | 231  51 | 273  49 | -55 |
| Emergency department^d^  *n reported* | 505  15 | 815  15 | -406 |
| Hospital admissions  *n reported* | 1,612  18 | 2,437  24 | -1,082 |
| Additional health care costs^e^  *n reported* | 1,693  40 | 1,991  45 | -391 |
| **Total health care costs** | **12,248** | **16,055** | **-3,807 (-6,580 to -1,127)** |
|  |  |  |  |
| Absenteeism paid work^f^  *n reported* | 8,708  43 | 8,818  40 | -110 |
| Presenteeism paid work^f^  *n reported* | 2,434  25 | 560  18 | 1,874 |
| Absenteeism unpaid work^f^  *n reported* | 4,655  64 | 4,653  53 | 2 |
| **Total productivity losses**  **Total societal costs** | **15,797**  **28,046** | **14,031**  **30,086** | **1,767 (-5,358 to 8,373)**  **-2,674 (-19,603 to 5,547)** |

*Abbreviations: Confidence Interval (CI), Standard deviation (SD), number (n)*

*Values are presented as pooled estimated means in rounded Euros. ‘N reported’ are the number of patients that reported costs for a certain subcategory in the original data set.

^a^ Surgical costs include the price for the procedure, implants and haemostatics, and costs for days of admission to the hospital for the initial intervention.

^b^ Other primary care includes visits to social workers, occupational therapists, speech therapists, dieticians, alternative therapists and psychologists.

^c^ Care at home was not reported by any of the patients in the posterior group, to note is that the reported costs are estimated pooled means, using multiple imputation and bootstrapping.

^d^ Emergency department includes visits to the emergency department and transportation to the hospital by ambulance.

^e^ Additional health care costs include day-care or admissions to a health institution other than a hospital, rehabilitation centre or psychiatric institution.

^f^ Absenteeism is the amount of days that a patient was absent from work times the Dutch reference wage per day. The ‘friction cost method’ was applied, assuming that after a period of 85 days of complete absence, an employee gets replaced by another worker, and productivity would be restored. Presenteeism is the loss of productivity by patients that were not able to fully fulfil their jobs. It was calculated by the proportion of productivity loss of a general working day, times the duration in days, times the average wage per day. Unpaid work is the loss of productivity during unpaid activities such as household work and volunteering. It was calculated by hours a day, times the duration in days, times the Dutch reference price for one hour of unpaid work.

**
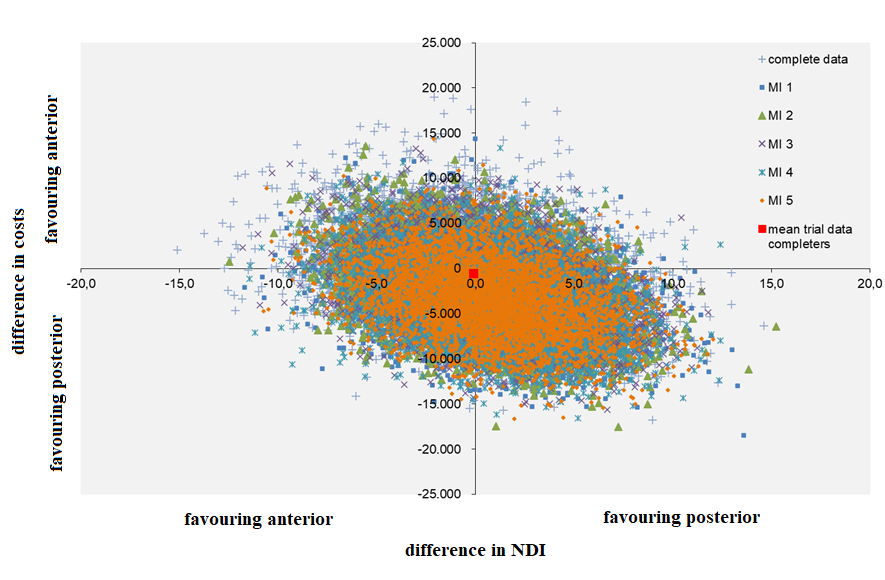
Figure 1.** **Incremental Cost-Effectiveness Plane comparing Cost Difference and Neck Disability Index from a Societal Perspective.**

*Abbreviations: Multiple imputation sets (MI).*

Figure 1 depicts the cost difference of posterior versus anterior surgery on the vertical axis in Euros, and the difference in Neck Disability Index (NDI) score between groups on the horizontal axis.

**
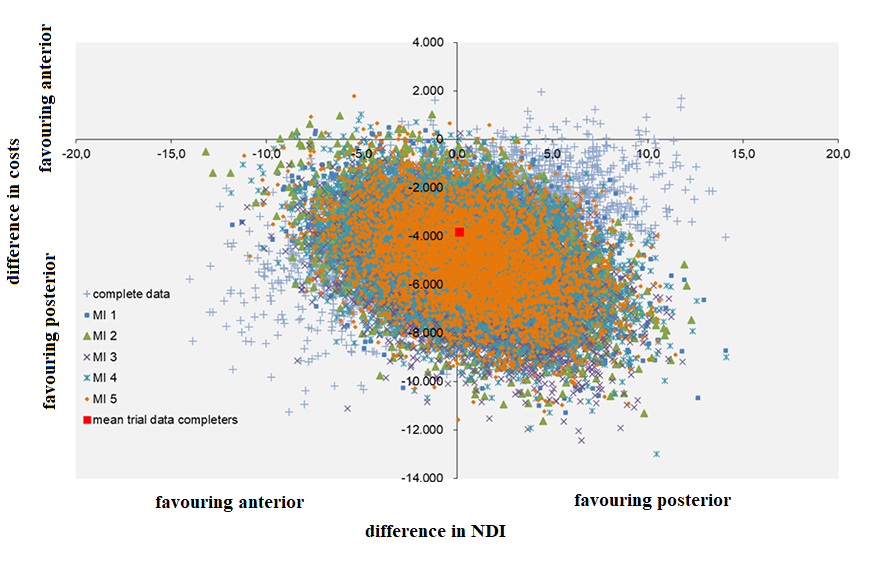
Figure 2. Incremental Cost-Effectiveness Plane comparing Cost Difference and Neck Disability Index from a Healthcare Perspective.**

*Abbreviations: Multiple imputation sets (MI).*

Figure 2 depicts the cost difference of posterior versus anterior surgery on the vertical axis in Euros, and the difference in Neck Disability Index (NDI) score between groups on the horizontal axis.

**
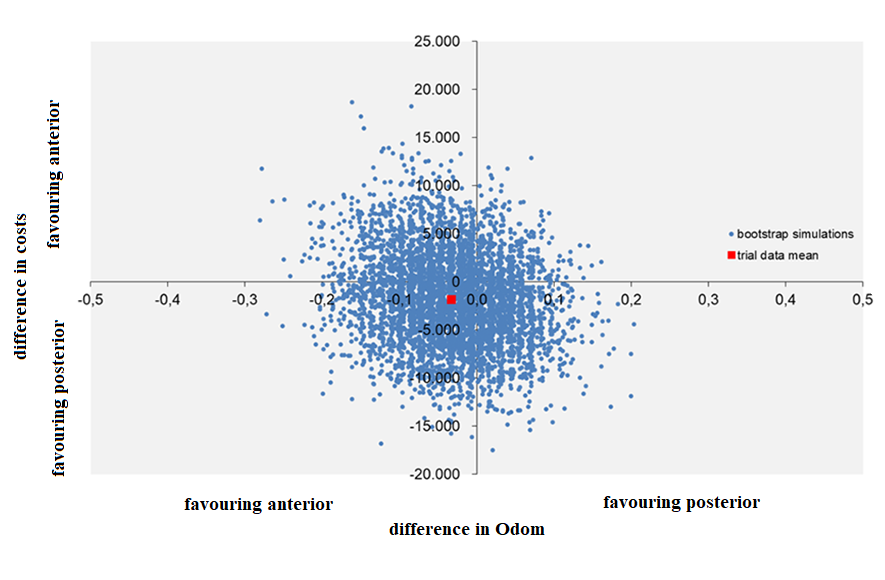
Figure 3. Incremental Cost-Effectiveness Plane comparing Cost Difference and Odom Criteria from a Societal Perspective.**

Figure 3 depicts the cost difference of posterior versus anterior surgery on the vertical axis in Euros, and the difference in proportion of Successful Odom Scores between groups on the horizontal axis. A successful Odom score is the proportion of patients graded ‘excellent’ or ‘good’ on the modified Odom criteria, based on patients with complete cost data for all time points and an available Odom score (n=80 in the posterior group and n=73 in the anterior group).

**
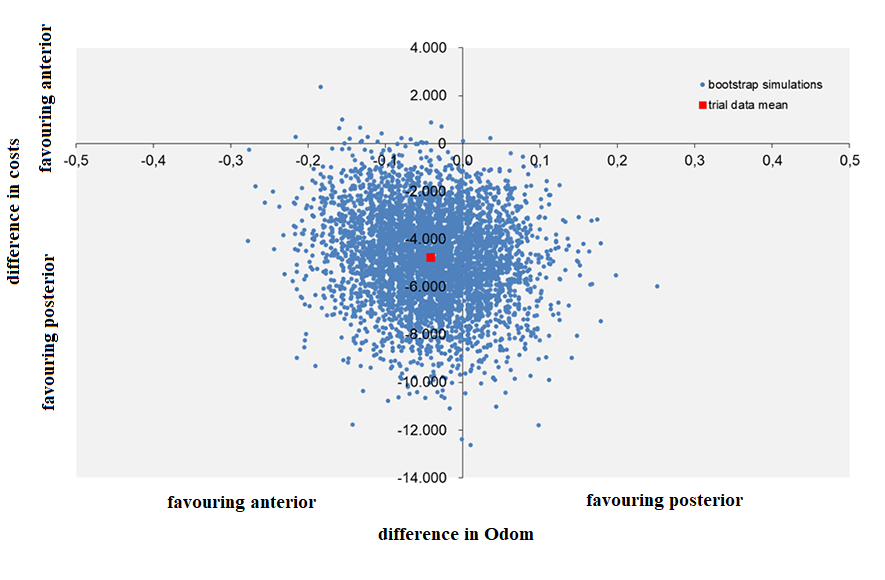
Figure 4. Incremental Cost-Effectiveness Plane comparing Cost Difference and Odom Criteria from a Healthcare Perspective.**

Figure 4 depicts the cost difference of posterior versus anterior surgery on the vertical axis in Euros, and the difference in proportion of Successful Odom Scores between groups on the horizontal axis. A successful Odom score is the proportion of patients graded ‘excellent’ or ‘good’ on the modified Odom criteria, based on patients with complete cost data for all time points and an available Odom score (n=80 in the posterior group and n=73 in the anterior group).
